# The Schistosome Oesophageal Gland: Initiator of Blood Processing

**DOI:** 10.1371/journal.pntd.0002337

**Published:** 2013-07-25

**Authors:** Xiao-Hong Li, William de Castro-Borges, Sophie Parker-Manuel, Gillian M. Vance, Ricardo DeMarco, Leandro X. Neves, Gareth J. O. Evans, R. Alan Wilson

**Affiliations:** 1 National Institute of Parasitic Diseases, Chinese Center for Disease Control and Prevention, Key Laboratory of Parasitology and Vector Biology, Ministry of Health, Shanghai, People's Republic of China; 2 Centre for Immunology and Infection, Department of Biology, University of York, York, United Kingdom; 3 Laboratório de Enzimologia e Proteômica, Instituto de Ciências Exatas e Biológicas – ICEB, Departamento de Ciências Biológicas – DECBI, Universidade Federal de Ouro Preto, Ouro Preto, Minas Gerais, Brasil; 4 Departamento de Física e Informática, Instituto de Física de São Carlos, Universidade de São Paulo, São Carlos, São Paulo, Brazil; McGill University, Canada

## Abstract

**Background:**

Although the ultrastructure of the schistosome esophageal gland was described >35 years ago, its role in the processing of ingested blood has never been established. The current study was prompted by our identification of MEG-4.1 expression in the gland and the observation of erythrocyte uncoating in the posterior esophagus.

**Methodology/Principal Findings:**

The salient feature of the posterior esophagus, characterized by confocal and electron microscopy, is the enormous increase in membrane surface area provided by the plate-like extensions and basal invaginations of the lining syncytium, with unique crystalloid vesicles releasing their contents between the plates. The feeding process was shown by video microscopy to be divided into two phases, blood first accumulating in the anterior lumen before passing as a bolus to the posterior. There it streamed around a plug of material revealed by confocal microscopy as tethered leucocytes. These were present in far larger numbers than predicted from the volume of the lumen, and in varying states of damage and destruction. Intact erythrocytes were detected in the anterior esophagus but not observed thereafter, implying that their lysis occurred rapidly as they enter the posterior. Two further genes, MEGs 4.2 and 14, were shown to be expressed exclusively in the esophageal gland. Bioinformatics predicted that MEGs 4.1 and 4.2 possessed a common hydrophobic region with a shared motif, while antibodies to SjMEG-4.1 showed it was bound to leucocytes in the esophageal lumen. It was also predicted that MEGs 4.1 and 14 were heavily O-glycosylated and this was confirmed for the former by 2D-electrophoresis and Western blotting.

**Conclusions/Significance:**

The esophageal gland and its products play a central role in the processing of ingested blood. The binding of host antibodies in the esophageal lumen shows that some constituents are antibody targets and could provide a new source of vaccine candidates.

## Author Summary

Host blood ingested by adult schistosomes resident in the bloodstream, passes down a short esophagus to reach the gut where proteolysis of its constituents occurs. The esophageal gland surrounds the posterior half of the esophagus, which has a membrane surface area enormously expanded into plates that must be crucial for its functioning. The contents of a unique crystalloid vesicle, manufactured in the gland, are released into the lumen and accumulate between the plates. Incoming host leucocytes are tethered in the lumen of the posterior esophagus and damaged or destroyed there. Erythrocytes are also lysed as they enter the posterior esophagus. We have identified two further protein products of the gland, to add to the two we previously described, which may potentially interact with the incoming host cells to determine their fate. Finally, we have shown the antibodies from infected hosts can recognise and bind to the esophageal gland secretions. This raises the possibility that gland constituents may serve as a new source of candidates for both a therapeutic and prophylactic vaccine.

## Introduction

Although adult schistosomes feed on host blood, whilst residing in the bloodstream, little is known about the initial stages of the process as blood passes down the esophagus. This short tube connecting the oral cavity to the gut was originally shown to be lined by an extension of the syncytial tegument that covers the entire parasite [1], its junction with the gut epithelium (gastrodermis) being clearly demarcated by a septate desmosome [1], [2]. The mass of cells around the posterior esophagus, collectively referred to as the esophageal gland, was explored by electron microscopy in the 1970s but has been almost completely neglected since then [3]. The esophageal lining has a more highly specialised surface architecture than the normal tegument. Its cytoplasm in the anterior half contains characteristic tegumental discoid bodies and multilaminate vesicles [4], the latter replaced by a distinct secretory inclusion in the posterior half. The cell bodies, where the various inclusions originate, are numerous in the anterior esophagus [2] and even more closely packed in the posterior [1], [5]. The inclusions in the posterior esophagus [3] contain a crystalline core arranged in parallel layers and sometimes circular profiles; hereafter we refer to them as ‘crystalloid vesicles’. Their role is presently a matter of conjecture.

The process of blood feeding has received scant attention, only a brief verbal description noting the opening and closing of the oral cavity every 1–2 seconds [6]. Additionally a single micrograph in a transmission electron microscopy (TEM) study of the worm gut revealed the presence of intact erythrocytes and platelets in the lumen of the anterior esophagus [7]. The one pertinent quantitative study involved the use of Cr^51^-labelled erythrocytes to compare the kinetics of blood ingestion in vivo, by male and female worms [8]. Lastly, as part of a proteomic analysis of worm vomitus, we observed the uncoating of PKH2-labelled erythrocytes as they passed down the esophagus, with the transfer of the lipophilic dye to the lining; this provides direct evidence for the interaction of gland products with incoming blood [9]. Little is currently known about the molecular products of the gland. The first constituent localised there, by whole mount in situ hybridisation (WISH; [10]) and immunocytochemistry [11], was SmMEG-4.1 (previously known as Ag10.3; [12]) encoded by a microexon gene (MEG). More recently a member of the SmVAL family, SmVAL-7, was exclusively localised to the gland by WISH [13]. Lastly, the reactivity of the gland for labelled peanut agglutinin, which binds to O-glycans, has been demonstrated [14]. The above descriptions refer entirely to *S. mansoni*, nothing similar having been reported for *S. japonicum*.

In this paper we use confocal microscopy to investigate the detailed morphology of the esophagus and the associated cells of *S. mansoni* and *S. japonicum*, with additional TEM and scanning electron microscope (SEM) observations. We have analyzed the blood feeding process by video microscopy and determined the distinct pathways for processing of blood components. We have identified further specific molecular constituents of the esophageal gland and used bioinformatics to predict their properties. For one of the proteins we have confirmed these predictions by Western blotting. Finally, we demonstrated the antigenicity of the gland secretions, indicating their potential as new vaccine candidates.

## Materials and Methods

### Ethics statement

#### York

Procedures involving animals were carried out in accordance with the UK Animals (Scientific Procedures) Act 1986, and authorised on personal and project licences issued by the UK Home Office. The study protocol was approved by the Biology Department Ethical Review Committee at the University of York.

#### Ouro Preto

The protocol for maintenance of the *S. mansoni* life cycle was reviewed and approved by the local ethics committee on animal experimentation, Comissão de Ética no Uso de Animais (CEUA), Universidade Federal de Ouro Preto (UFOP), and received the protocol no. 2011/55.

#### Shanghai

Animal care and all animal procedures were carried out in compliance with the Guidelines for the Care and Use of Laboratory Animals produced by the Shanghai Veterinary Research Institute. The study was approved by the Ethics Committee of the Institute of Parasitic Diseases, Chinese Center for Disease Control and Prevention.

### Parasite maintenance and worm recovery

#### York

A Puerto Rican isolate of *S. mansoni* was maintained using albino *Biomphalaria glabrata* snails and NMRI strain mice as laboratory hosts, the latter being exposed to 200 cercariae. For some analyses worms were fixed *in situ* in the portal system of anaesthetised or freshly killed mice by injection of paraformaldehyde fixative (PF: 4% paraformaldehyde in 0.1 M phosphate buffer saline) either down the superior mesenteric vein or backwards through the liver from the hepatic vein. Fixed worms were flushed out of the system with medium and further fixed for 4 hours in PF.

#### Ouro Preto

Swiss mice bred in-house and weighing approximately 20 g were infected percutaneously with 250 cercariae of *S. mansoni* of the LE isolate.

#### Shanghai

Cercariae of *S. japonicum* were shed from naturally infected *Oncomelania hupensis* snails collected from fields in Anhui Province, P.R. China. Rabbits and mice were experimentally infected percutaneously with 1,000 or 40 cercariae, respectively.

In all three institutions adult parasites were obtained by portal perfusion of hosts, at 5–6 weeks after infection for *S. japonicum* and 4–7 weeks for *S. mansoni*, using RPMI-1640 medium (minus phenol red) buffered with 10 mM HEPES (both from Invitrogen, Paisley, UK). Parasites were extensively washed in the same medium and tissue debris and any damaged individuals removed under a dissecting microscope.

### Immunocytochemistry

Both *S. mansoni* and *S. japonicum* worms were prepared for morphological analysis by fixation and storage in AFA (ethanol/40% formaldehyde/glacial acetic acid, in the ratio 85∶10∶5), staining for 30 minutes in Langeron's Carmine [15], differentiation in 70% acid ethanol until background stain had disappeared, clearing, and mounting in DPX (VWR International Ltd, Lutterworth, UK). For immunocytochemistry, intact adult worms were fixed and permeabilized using the protocol described by Mair et al. [16]. Briefly, they were fixed for four hours in 4% PF and then incubated in permeablising fluid (PBS containing 0.1% Triton x-100, 0.1% BSA and 0.02% sodium azide; antibody diluent solution; AbD) overnight at 4°C. Subsequent steps were carried out, with shaking, at 4°C in AbD. For visualization of the musculature by staining of f-actin, permeabilised worms were incubated overnight with a 1∶100 dilution of AF555-conjugated phalloidin (Invitrogen, Molecular Probes). For staining of nuclei, permeabilised worms were incubated with 4′,6-diamidino-2-phenylindole (DAPI;1 µg/ml in PBS; Sigma-Aldrich, Poole, Dorset, UK) for 30 minutes. The reactivity of permeabilised worms with peanut agglutinin (PNA ) was determined using Fluorescein isothiocyanate (FITC)-conjugated lectin from *Arachis hypogaea* ( Sigma-Aldrich) at a concentration of 0.01 mg/ml for two days (after Collins et al. [14]). In order to investigate the localisation of SjMEG-4.1 an antibody was raised by vaccinating rats with a synthetic peptide (CEGDFYELEPPVHYYD) derived from the parent protein sequence and coupled via the terminal cysteine to carrier ovalbumin. A 100 µg sample, emulsified in 100 µl complete Freund's adjuvant (Sigma-Aldrich), was administered subcutaneously to rats on the back of the neck, with two subsequent boosts at 3 weeks intervals with conjugates emulsified in incomplete Freund's adjuvant, before a terminal bleed at week 8. Permeabilized *S. japonicum* worms were reacted with the rat anti-SjMEG-4.1 antibody at 1∶1000 dilution in AbD containing 10% normal goat serum and given extensive washes in AbD before localization using Alexa Fluor (AF) 488-labeled goat anti-rat antibody at 1∶200 dilution (Sigma-Aldrich).

Worms recovered from mice and hamsters were used to detect the presence of antibodies in the esophagus lumen, using two host-specific antibodies, each also serving as the control for the alternate species. Mouse antibodies were probed using FITC-labelled goat anti-mouse polyvalent immunoglobulins (G, A, M; Sigma-Aldrich) and hamster antibodies using AF488-labeled goat anti-hamster IgG (H+L), absorbed against mouse and rat IgG prior to conjugation (Invitrogen). Permeabilized worms were incubated with labelled antibodies for two days at 4°C, washed several times in AbD and then counterstained with phalloidin as above.

### Confocal microscopy

Optical slices were obtained using a LSM-710 confocal microscope (Zeiss, Cambridge, UK). Imaging conditions were as follows: DAPI: 405 nm diode laser with 405 main beam splitter (MBS); FITC/AF488: 3 mW argon laser with 488/561/633 MBS; Phalloidin AF555: 561 nm diode laser with 488/561 MBS; Langeron's carmine: 561 nm diode laser with 488/561 MBS. The number of leucocytes tethered in the posterior esophagus lumen of Langeron's-stained males and females was estimated from consecutive images in a Z stack, taken at 1 µm intervals over approximately 28 µm, encompassing the full depth. Z stacks for counting the number of cells in anterior and posterior cell masses were obtained using either the LSM-710 microscope or the LSM-780 multiphoton mounted on a Axio Observer.Z1 invert microscope (Zeiss), operating with a Chameleon pulsed Ti∶Sa IR Laser (Coherent, Santa Clara, CA, USA).

### Scanning and transmission electron microscopy

The structure of the esophagus in both *S. mansoni* and *S. japonicum* was examined by TEM. Worms in the act of feeding both in vitro and in vivo were also examined. They were fixed overnight at 4°C in 2.5% glutaraldehyde/4% paraformaldehyde in 100 mM phosphate buffer, pH 7.4. Following two washes in phosphate buffer, they were post-fixed in osmium tetroxide for 2 hours, washed in H_2_O and dehydrated through a water-acetone series before single embedding in Spurr's resin. Worm heads were then cut out of the resin and mounted in a transverse or longitudinal orientation on plastic stubs. Thick sections (0.5 µm) were cut and stained with toluidine blue for examination on a light microscope to identify the presence of the anterior/posterior esophagus. Thin sections (70 nm) were then cut onto grids and post-stained with saturated uranyl acetate in 50% ethanol, and Reynolds' lead citrate solution before examination in a Tecnai 12 BioTwin microscope (FEI, Hillsboro, OR, USA). Measurements of structures in TEMs were performed using the ‘Analyzingdigitalimages’ package (Lawrence Hall of Science, UC Berkeley, CA, USA) with the intrinsic scale as calibrator.

The luminal surface of the esophagus was also examined by SEM to determine its architecture. Adult *S. mansoni* were fixed in Baker's formaldehyde calcium overnight at room temperature and the head was then sliced down the longitudinal axis. The halves were washed, dehydrated, mounted on stubs with the lumen uppermost, sputter coated with 7 nm gold/palladium and examined using a JSM 6490-LV microscope (Jeol, Tokyo, Japan).

### In vitro feeding experiments

The process of blood feeding was recorded at 37°C on a compound microscope with 10× to 40× objectives and resulting movies subjected to frame analysis. Worms perfused from mice at 7 weeks post-infection were cleaned of debris, washed several times using RPMI 1640 medium (Invitrogen) and distributed 1–2 per well in a 24-well culture plate. Before filming a few drops of a 1% suspension of washed mouse erythrocytes was added to a test well and the cells allowed to settle. Time lapse images of the worms were acquired at 5–16 frames/s by a Rolera-XR CCD camera (QImaging, Surrey, BC, Canada) through the air objective of a Nikon TE200 inverted microscope under brightfield illumination. Camera control and image acquisition were coordinated by SimplePCI, version 6.5, software (Hamamatsu Corporation, Sewickley, PA, USA). Sequential images were examined using ImageJ (available from NIH, Bethesda, MD, USA) and Imagistik Image Viewer (Informatik Inc., Devon, PA, USA) to select representative feeding activities. Calibration scales at each magnification, plus single images were exported as tif files for size measurements on esophageal features using the ‘Analyzingdigitalimages’ package (Lawrence Hall of Science). Movies were generated using QuickTime 7 Pro (Apple Inc., Infinite Loop, Cupertino, CA, USA), trimmed to correct size, and exported in MP4 format.

### Bioinformatic analysis

Previously we analyzed changes in gene expression during infection of the mammalian host by *S. mansoni*, using a genome-wide microarray [17], by grouping datasets according to putative function. Here we have reanalysed the complete dataset to highlight those genes most highly upregulated between the intramolluscan germ ball and day three schistosomulum stages, irrespective of their gene ontology, focusing on the top twenty (Table S1). In addition to SmMEG-4.1 and SmVAL-7, the two known constituents of the esophageal gland, seven other MEG transcripts were prominent and two, MEG-14 and MEG-4.2, were selected for more detailed investigation in both *S. mansoni* and *S. japonicum* together with the MEG-4.1 of *S. japonicum*. Sequences for homologues of these *S. mansoni* genes in *S. japonicum* and *S. haematobium* were obtained by doing a blastp search against the NCBInr database (GenBank), with the exception of the *S. haematobium* sequence for MEG-4.2 which was obtained by a tblastn search using the *S. mansoni* protein as query against the database of assembly reads from a RNAseq library available at http://bioinfosecond.vet.unimelb.edu.au/S_haematobium_060212/ [18].

Gene-finding programmes have a limited capacity to predict the sequence of MEG genes accurately; hence the selected sequences were submitted to BLAST searching on the NCBInr database to obtain the longest transcript. The presence of a signal peptide, N and O glycosylation sites and transmembrane regions were predicted using SignalP, NetNGlyc, NetOGlyc and TMHMM programmes, respectively, all available at http://www.cbs.dtu.dk/services. Parker hydrophilicity plots were performed using http://tools.immuneepitope.org/tools/bcell/iedb_input available at www.iedb.org. Multiple sequence alignments were conducted using Clustal X2, version 2.1. S.

### Whole mount in situ hybridisation (WISH)

Segments of SmMEG-4.2 and 14 sequences were amplified with cDNA produced from schistosomula as the template, using the following primers:

- MEG-4.2 forward ATGAATTTCTTGACACTTTACGTAACT,
- MEG-4.2 reverse TGAAGTAATATGATATAGCTCTTGGAA,
- MEG-14 forward ATGAATAGGTTCTTTTGGACTGTCA,
- MEG-14 reverse TACGATAGGGACAGCCGC.

Gene segments were cloned into pGEM-T Easy (Promega, Southampton, UK) and digoxigenin-labelled RNA probes synthesised as reported previously [10]. Adult worms were fixed in Carnoy's fixative (ethanol∶chloroform∶glacial acetic acid in a 6∶3∶1 ratio) for 2 hours on ice, with shaking. After two washes of 5 minutes in absolute ethanol they were subjected to one hour's fixation in MEMFA (0.1 M MOPS, 2 mM EGTA, 1 mM MgSO4, 3.7% formaldehyde in water) at room temperature. After two more washes in ethanol, they were stored under ethanol at −20°C.

WISH was carried out according to the method of Dillon et al., 2007 [10]. Briefly, worms were permeablised using proteinase K (Roche Applied Science, Burgess Hill, Kent, UK). After alkylation and refixation in 10% formalin in PBS with 0.1% Tween 20 (PBSAT), the worms were incubated in hybridisation buffer for 2 hours at 60°C. Digoxigenin-labelled RNA probe was added to fresh hybridisation buffer, and the hybridisation step was carried out overnight at 60°C. After several washes of increasing stringency, the worms were washed twice in maleic acid buffer (MAB; 100 mM maleic acid, 150 mM NaCl, pH 7.8, 0.1% Tween 20) before incubation in blocking solution composed of MAB with 2% blocking reagent (Roche) and 20% heat-treated lamb serum for two hours at room temperature. Alkaline phosphatase-conjugated anti-digoxigenin FAb fragments (Roche) were added at 1∶2000 dilution in blocking solution, and the specimens were incubated overnight at 4°C with shaking. Following several washes, the worms were incubated in NBT/BCIP solution (NBT: nitro-blue tetrazolium chloride; BCIP: 5-bromo-4-chloro-3′-indolyphosphate p-toluidine salt; Roche) and observed as colour developed. When clear results were obtained, the worms were washed twice in PBSAT, and then fixed in 10% formalin in PBSAT. Results were documented using a Leica DM2500 microscope attached to an 18.2 colour mosaic camera with SPOT Advanced software (SPOT Imaging Solutions, Diagnostic Instruments, Sterling Heights, Michigan, USA).

### Worm head preparation, two-dimensional electrophoresis (2DE) and Western blotting

The heads of approximately 1600 male *S. mansoni* were excised using a sterile blade, rapidly immersed in ice-cold 25 mM Tris-HCl buffer, pH 7.5, containing 1 mM dithiothreitol (DTT) plus Protease Inhibitor Cocktail (Sigma-Aldrich) and sonicated for extraction of the soluble protein fraction. The homogenate was centrifuged at 20,000× *g* for 30 min at 4°C, the supernatant discarded and the membranous pellet washed out in Tris-HCl buffer to remove any remaining soluble proteins. After a new round of centrifugation as above, the resulting pellet was sonicated in rehydration solution (7 M urea, 2 M thiourea, 4% CHAPS (3-[(3-Cholamidopropyl) dimethylammonio]-1-propanesulfonate)) and re-centrifuged at 20,000× *g* at 10°C to recover membrane-associated proteins in soluble form.

Approximately 50 µg of protein preparation were diluted in fresh rehydration solution to a final volume of 125 µL, containing 1% DTT plus 0.8% ampholytes (IPG buffer pH 3–10). The sample was electro-focused in the IPGPhor (GE, Healthcare, Chalfont St Giles, UK) using linear IPG-strips (7 cm, pH 3–10) according to the following protocol: 12 hours of passive gel rehydration, 30 minutes at 500 V, 30 minutes at 1000 V and additional 3 hours at 8000 V. Focused proteins were first reduced and then alkylated in equilibration buffer (75 mM Tris-HCl pH 8.8, 6 M urea, 30% glycerol, 2% SDS, 0.002% bromophenol blue) containing 1% DTT or 2.5% iodoacetamide, respectively, 15 minutes for each step. The equilibrated IPG strip was separated across an 8×10×0.1 cm 12% SDS-PAGE gel, run at a constant 100 V until the dye left the gel (approximately 2.5 hours), fixed in 40% ethanol:7% acetic acid (v/v H_2_O) for 30 minutes and finally stained in Coomassie reagent.

Two replicate gels, obtained as described above, were produced and immediately transferred to two polyvinylidene difluoride (PVDF) membranes on a miniVE blot module (Hoefer, Holliston MA, USA) at 200 µA over 2 hours, using 25 mM Tris-HCl buffer, pH 8.8, containing 192 mM glycine, 20% ethanol, 0.02% SDS. One membrane was blocked overnight at room temperature in 50 mM Tris-HCl buffer, pH 7.5 (TBS) plus 0.3% Tween 20 and 5% non-fat milk powder, prior to detection of SmMEG-4.1. After blocking, the membrane was washed 3×5 min, in 10 mM Tris-HCl buffer pH 7.5 and then incubated in immunoblotting buffer (TBS plus 100 mM NaCl, 0.05% Tween 20) containing 5% non-fat milk. Anti-SmMEG4.1 serum produced in rats [11] was added to a final dilution of 1∶2000 and allowed to react for 3 hours at room temperature. Horseradish peroxidase (HRP)-conjugated anti-rat IgG (1∶2000) was employed to detect the target protein using Enhanced Chemiluminescence (ECL) Plus Western Blotting Detection Reagents (GE Healthcare). For the second membrane, *Peanut* agglutinin (PNA)-binding proteins were detected using the DIG Glycan Differentiation kit (Roche) as per manufacturer's instructions. Briefly, the PVDF membrane was incubated in solution (TBS plus 150 mM sodium chloride) containing 10% blocking reagent, for 2 hours. After several washes in TBS, digoxigenin-labelled PNA lectin in 0.01 mg/ml concentration was added and incubated for 1 hour at room temperature in TBS supplemented with 1 mM MgCl_2_, MnCl_2_ and CaCl_2_. After a 1 hour incubation with anti-digoxigenin alkaline phosphatase-labelled antibody the colour was developed using NBT/BCIP substrate (Sigma-Aldrich).

## Results

### The oesophagus has a complex, highly organised structure

**Figure 1:**
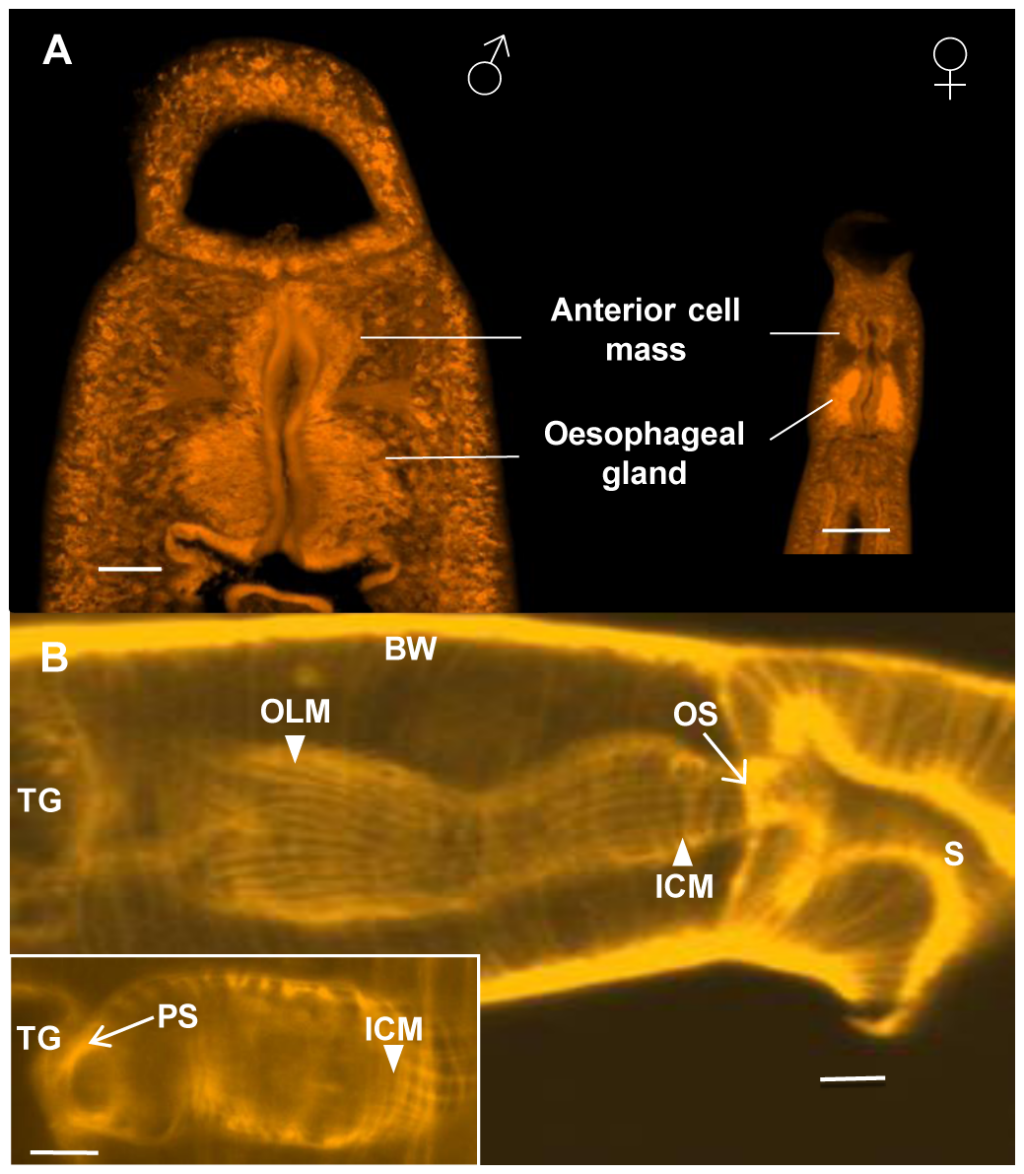
Layout of the esophageal region and its musculature. (A) To-scale confocal images of *S. japonicum* adult male (left) and female (right) stained with Langeron's carmine, to illustrate the large discrepancy in size of their esophageal glands. (B) A longitudinal side view of a female *S. mansoni*, stained with phalloidin to show only the distribution of F-actin in muscles. The minute inner circular (ICM) and outer longitudinal muscle fibers (OLM) that invest the syncytial esophageal lining appear as a fine meshwork. In comparison the larger circular and longitudinal fibers of the body wall (BW) and oral sucker (S) are intensely stained. An oral sphincter (OS, in side view) comprising a stronger circular fiber is visible at the junction between oral cavity and esophagus; a posterior sphincter (PS) is present at the junction between the esophagus and the transverse gut (TG) (inset, en face view). Scale bars: A, 50 µm; B and inset, 10 µm.

Obtaining a representative image of the anterior region of a schistosome was problematic because all fixation regimes caused some degree of contraction and distortion. This is very evident when live male worms are compared with fixed worms (Table S2). In life the oesophagus is a straight tube while in death it has a sinusoidal appearance due to fixative-induced contraction. There is a clear demarcation into anterior and posterior compartments comprising roughly spherical masses of cells, the anterior being approximately one third the volume of the posterior (Figure 1A; Table S2). Due to size disparities between the sexes, the esophageal region of the male is much larger than that of the female (Figure 1A; Table S2). The esophageal lining is invested by a muscular network comprising inner circular and outer longitudinal fibres (Figure 1B; Table S2), the spacing between which creates a lattice that works in unison to generate the peristalsis propelling food from mouth to intestine; two stronger circular fibres act as sphincters to control entry to and exit from the esophageal lumen (Figure 1B and inset). However, there is no obvious sphincter separating the anterior and posterior compartments. The esophagus empties into the most anterior region of the gut, approximately circular in cross section, lying transversely across the worm body (Figure 1A). From here two branches descend, either side of the reproductive organs, before uniting again as a single structure running towards the posterior.

**Figure 2:**
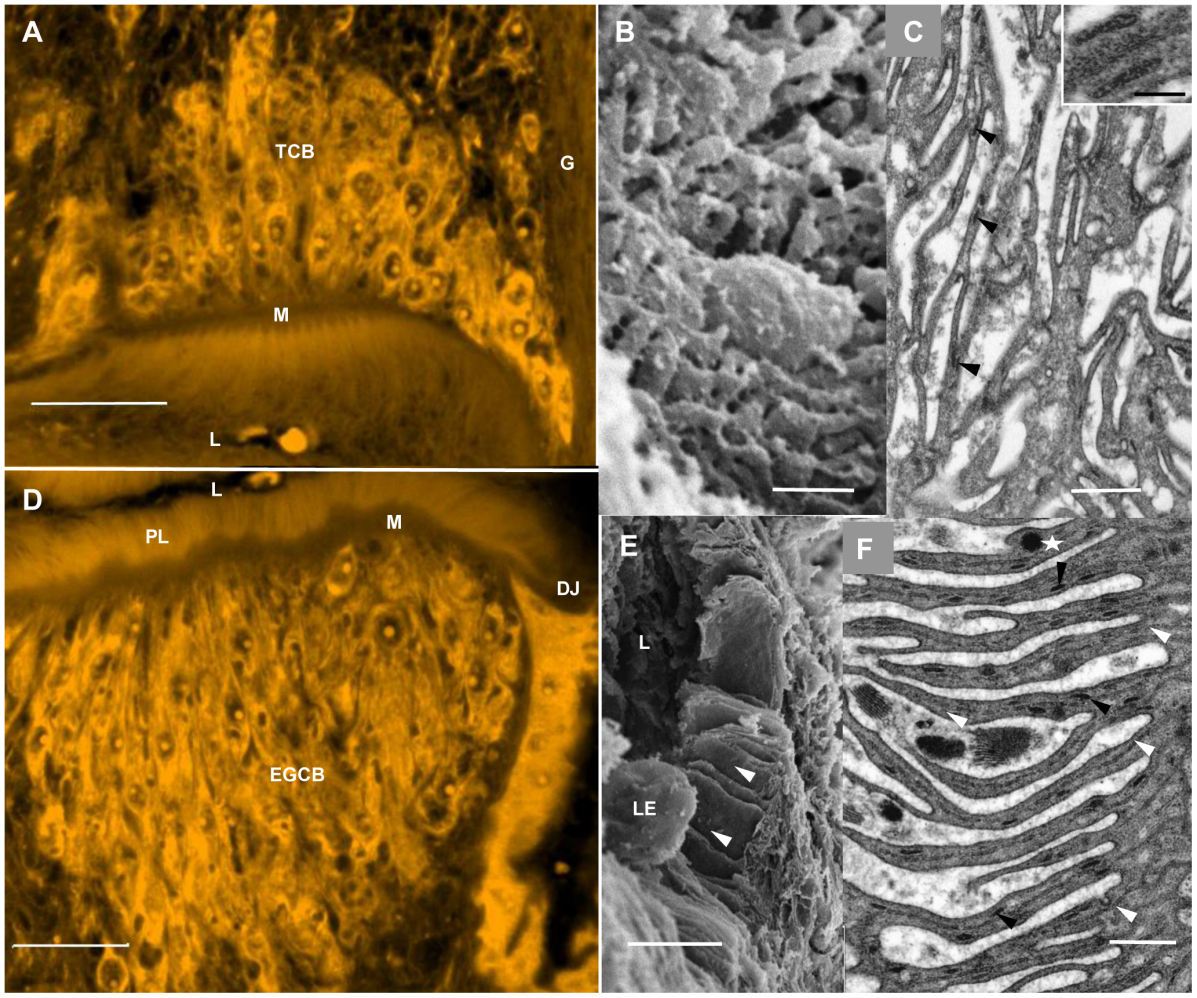
Three different views of esophageal cellular morphology. A–C, anterior esophagus; D–F, posterioresophageal gland. A and D show the whole region in optical section; B, C and E, F show only the respective esophageal linings at the much greater resolution provided by electron microscopy. (A) Confocal image of *S. japonicum* adult male stained with Langeron's carmine, showing arrangement of densely packed tegument cell bodies around the anterior esophageal lining; the musculature (M) is located as the dark line between the two. (L, Lumen; G, Central ganglion; TCB, tegument cell bodies). (B) SEM, anterior esophageal lining of *S. mansoni* showing its highly corrugated surface. (C) TEM, anterior esophageal lining of *S. mansoni* showing longitudinal orientation of the corrugations containing discoid bodies (arrowed and inset), typical of the tegument. (D) Confocal image of *S. japonicum* adult male stained with Langeron's carmine showing arrangement of cell bodies that comprise the esophageal gland around the posterior esophageal lining. (EGCB, esophageal gland cell bodies; PL, Plates; L, Lumen; M, Musculature; DJ, Desmosome junction between esophageal lining and gastrodermal epithelium.) (E) SEM of posterior esophageal lining of *S. mansoni* showing the luminal surface greatly extended to form thin plates (arrowed). (L, Esophageal lumen; LE, Leucocyte.) (F) TEM of the thin plates; a central double line (white arrows) is evident in each plate and discoid bodies (black arrows) are numerous. A single crystalloid vesicle (starred) is located close to a potential docking site. Scale bars: A, 50 µm; B, 1 µm; C, 500 nm; D, 50 µm; inset, 100 nm; E, 5 µm; F, 500 nm.

The anterior mass of cells is composed of morphologically typical tegument cell bodies exporting discoid bodies and multilaminate vesicles, but much more densely clustered than beneath the surface tegument (Figure 2A), giving the appearance of a glandular tissue. The approximate cell number was estimated as 750 in both *S. japonicum* and *S. mansoni* males from counts on consecutive slices in a Z stack, using DAPI-stained nuclei or Langeron's-stained nucleoli as indicators (Table S2; Movie S1). Another major distinction from the tegument is that the surface of the esophageal lining is highly corrugated (Figure 2B). Nevertheless, the longitudinal folds of cytoplasm contain only the typical tegumental inclusions such as discoid bodies (Figure 2C). In contrast, the lining and cell bodies of the posterior esophagus show significant modifications. In the confocal microscope (at the limit of resolution), the posterior lining displays shadowy striations (Figure 2D) which, when viewed in the TEM, are revealed as thin cytoplasmic extensions tapering towards their tips, and hereafter referred to as ‘plates’ (Table S2). The plates appear loosely separated, not tightly packed together as in previous descriptions [2]. SEM of worm slices reveals that they are orientated longitudinally, running in parallel from anterior to posterior of the gland (Figure 2E). They occasionally appear to bifurcate/merge, but we could not establish that individual plates ran the whole length. Each plate has two prominent central parallel lines formed by greatly extended invaginations of the basal syncytium membrane (Figure S1E; Table S2). The plates increase the luminal surface area of the posterior esophagus 26-fold, compared with its dimensions when treated as a smooth prolate spheroid.

**Figure 3:**
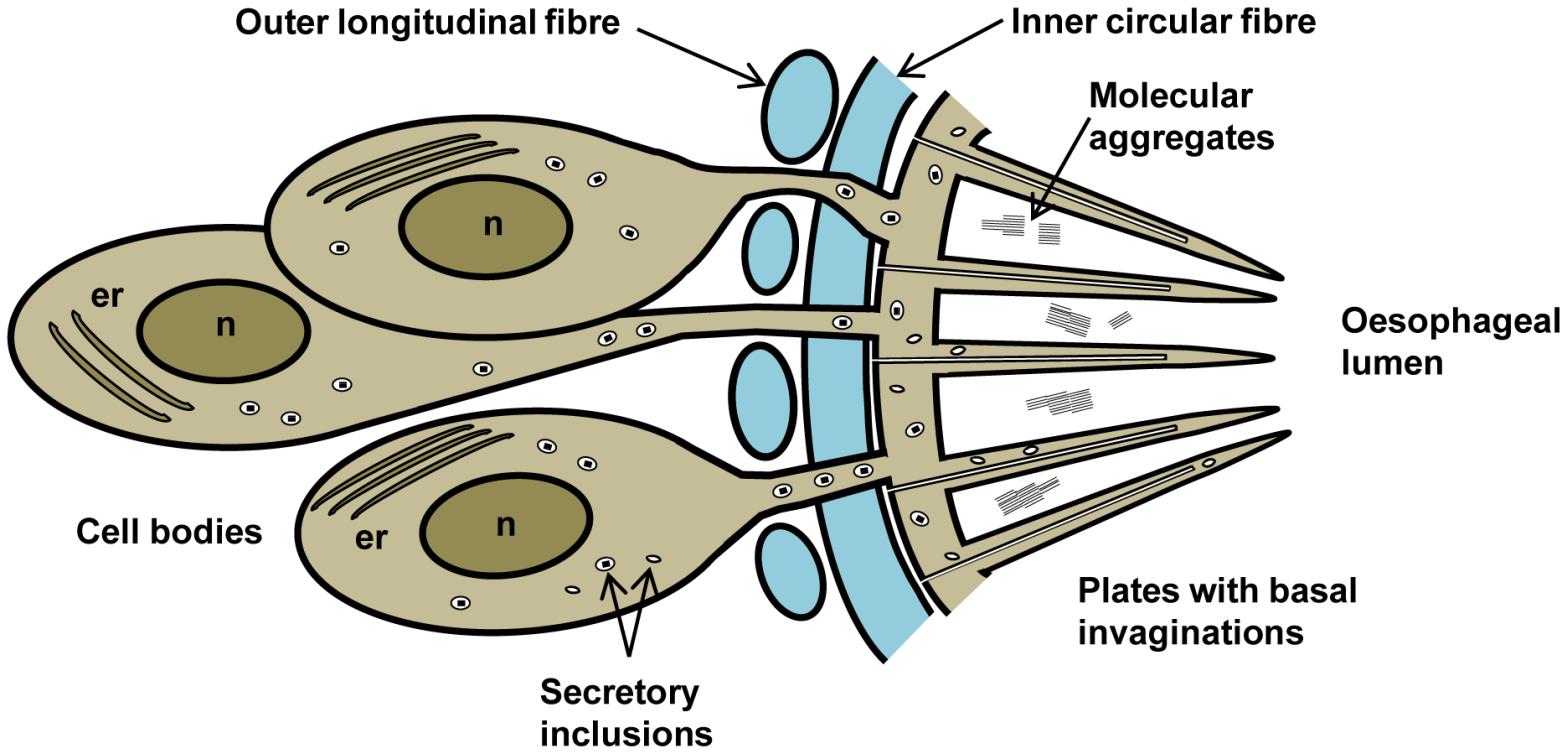
Diagrammatic representation of the esophageal gland in transverse section. A single cytoplasmic extension is shown running from each nucleated cell body, through the muscle layer to the lining syncytium, as we have no evidence that branching occurs, cf. tegument cell bodies. The secretory inclusions are crystalloid vesicles and discoid bodies.

The cell bodies of the posterior esophagus, which comprise the gland, may number as many as 1000 in *S. mansoni* and 1400 in *S. japonicum* males (Table S2; Movie S1). This close packing means some are positioned up to 80 µm from the lining syncytium but each is connected by microtubule-lined extensions, down which the secretory crystalloid vesicles travel in serried ranks (Figure S1B and D). The cell bodies are very obviously engaged in synthesis of protein for export, indicated by extensive rough endoplasmic reticulum (Figure S1A), prominent nucleoli (Figure S1B), Golgi bodies and assemblages of crystalloid vesicles in the cytoplasm (Figure S1C). Where the extensions from the cell bodies pass between the esophageal wall muscles, they are only just wide enough to accommodate a single crystalloid vesicle (Figure S1D). These vesicles contain parallel arrays of electron-dense material, in evenly spaced repeating units 19 µm apart, separated by an intervening electron-lucent layer (Figure S1C; Table S2). After the vesicles reach the thin lining syncytium, for the most part, they are confined to the base and a short distance up into the plates, while the typical tegumental discoid bodies appear capable of travelling much further towards the tips (Figure 2F; Figure S1E). The complex cellular architecture of the esophageal gland can be summarised in diagrammatic form (Figure 3).

### Blood feeding is a multi-step process

**Figure 4:**
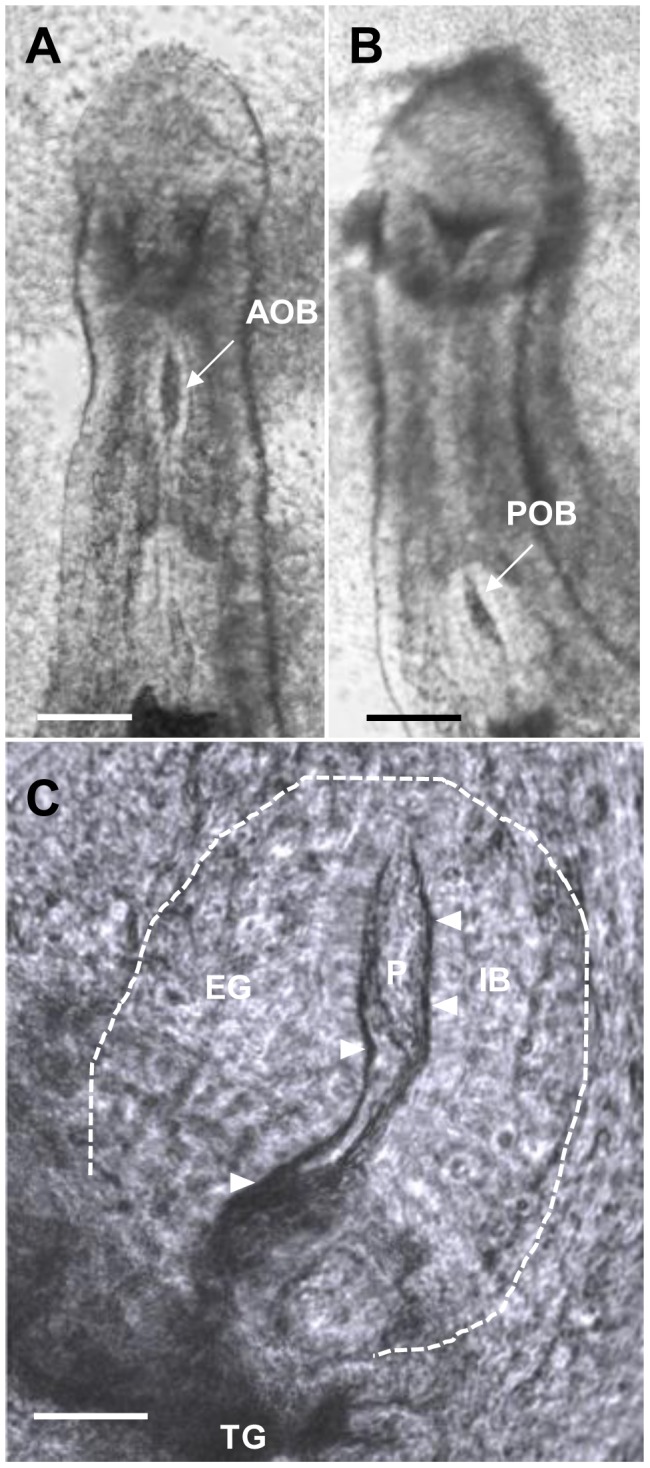
Frame analysis of the feeding process in *S. mansoni*. Single frame from movies of active male worms feeding in vitro on a dilute suspension of erythrocytes. (A) Blood accumulating in the lumen of the anterior esophagus to form a bulge (AOB). (B) The deflated anterior esophagus is replaced by a bulge in the posterior (POB), as blood transits. (C) Ingested blood (IB, arrowed) entering the lumen of the esophageal gland (EG, outlined, based on >40 consecutive images) flows as dark line around the plug (P) of material as it passes to the transverse gut (TG). A and B filmed at ×10 magnification, C at ×40. Scale bars: A & B, 100 µm; C, 25 µm.

We made a series of movies of male *S. mansoni* worms at 37°C to analyze the feeding process in detail. The initial step is a rapid grabbing motion of the oral sucker (4/sec, Movie S2). This is achieved when the sucker rim contracts, sweeping blood down into the oral cavity. The blood passes through the open oral sphincter to accumulate in the lumen of the anterior esophagus, which gradually expands to form a bulge (Figure 4A). In the second step, a wave of peristalsis passes down the esophagus driving the bolus to the posterior esophagus in a single smooth movement, as the anterior bulge deflates (Figure 4B; Movie S3). A second bulge is already apparent in the lumen of the posterior esophagus, reminiscent of a plug, which at high magnification appears to contain cells (Figure 4C). As the bolus arrives, this bulge expands with material streaming as a dark current around the plug (Movie S4). This dark material appears constrained within the lumen and does not enter the spaces between the plates. The ‘objects’ observed in the posterior esophageal plug appear to be stationary, not travelling with the flow of ingested blood as viewed in consecutive frames (Figure S3A). The final act in this process is the transit of ingested blood into the transverse gut, presumably when the posterior sphincter opens. It should be noted that although the gut empties via the esophageal lumen, a process of reverse peristalsis was not observed. Rather, vomiting was preceded by intense activity in the anterior gut, followed by relaxation of the esophageal wall that allowed a thin dark line of vomitus to travel towards the mouth (Figure S2A). This was followed by ejection of a stream of pigment particles from the oral sucker (Figure S2B).

We can estimate the volumes of the anterior and posterior bulges from their dimensions, taken from single video frames, using the formula for a prolate spheroid (Table S3). Given the cell content of a cubic mm of blood, the probable capacity at any instant in a male is 52 erythrocytes, 3 platelets and 0.07 leucocytes in the anterior bulge, and 125 erythrocytes, 7 platelets and 0.17 leucocytes in the posterior bulge. Video capture of females feeding was more difficult due to their small size and rapid movements so the estimate for the posterior bulge must be treated with caution, but equates to 25 erythrocytes, 1.5 platelets and 0.034 leucocytes (Table S3).

### Leucocytes are tethered in the posterior oesophagus

**Figure 5:**
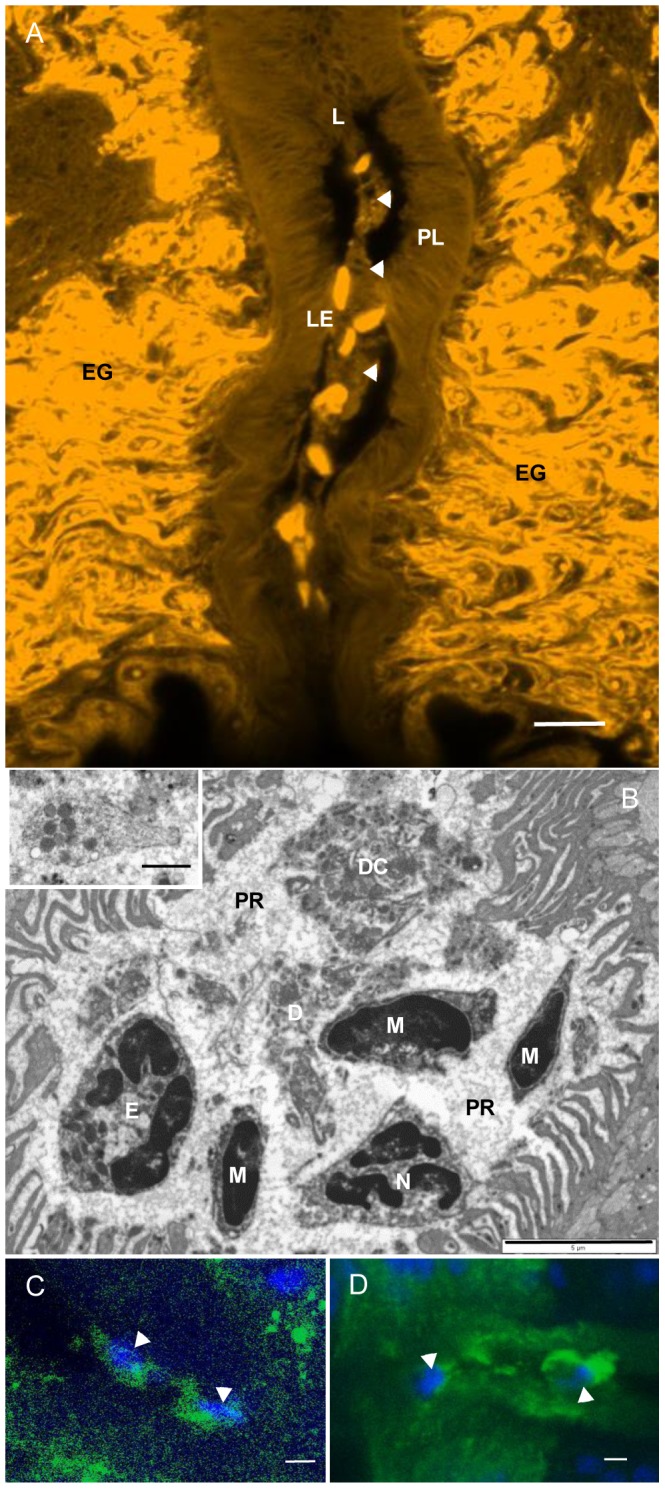
Leucocytes tethered in the posterior esophageal lumen. (A) Confocal image of adult male *S. mansoni* stained with Langeron's carmine, showing a string of host leucocytes (LE) tethered in the lumen (L), surrounded by extracellular material (arrowed). The esophageal plates (PL), at the limit of resolution, appear as fine striations. (B) TEM of posterior esophagus in *S. mansoni* showing damaged leucocytes (neutrophil, N; eosinophil, E; mononuclear cell, M; disintegrated cell, DC), a platelet with characteristic granules (inset) and debris (D) present in the centre of the lumen, surrounded by a dense precipitate (PR) of protein. (C) Localization of SjMEG-4.1 (green) in the esophageal lumen of a male *S. japonicum* strongly associated with the tethered leucocytes (blue, arrowed). (D) Ditto for a female *S. japonicum*. Scale bars: A, 10 µm; B, 5 µm; inset 1 µm; C & D, 5 µm.

When the stationary plug in the lumen of the posterior esophagus (Figure 4C and S3A), was examined by confocal microscopy it proved to be a string of host leucocytes trapped within extracellular material (Figure 5A). This interpretation was confirmed by TEM when the plug could be seen to comprise polymorphonuclear and mononuclear leucocytes in varied states of disintegration (Figure 5B); indeed, some were barely recognisable as leucocytes. In addition, occasional platelets were visible, identifiable by their size and complement of characteristic granules (Figure 5B, inset), while there was a granular matrix of precipitated proteins surrounding the cells and their debris. On occasion, the plug could be seen suspended in the centre of the lumen (Figure S3B), not in contact with the lining tissues. In contrast, other debris appeared to interact extensively with the edges of the plates (Figure S3B). A small number of damaged leucocytes reached the gut lumen, often in an advanced state of disintegration (Figure S3C and D). Z-stacks of consecutive confocal images through the lumen of the posterior esophagus permitted accurate estimation of the number of tethered leucocytes present, all determined on worms fixed in vivo to avoid in vitro artefacts. In the posterior esophageal lumen of male *S. mansoni* there was a mean of 28.6 (+S.E. 4.3; n = 15) leucocytes and in females, 10.36 (+S.E. 3.12; n = 11), numbers which greatly exceeded the theoretical predictions based on lumen volume and density of cells in blood (Table S3).

### Erythrocyte processing occurs in the posterior oesophagus

**Figure 6:**
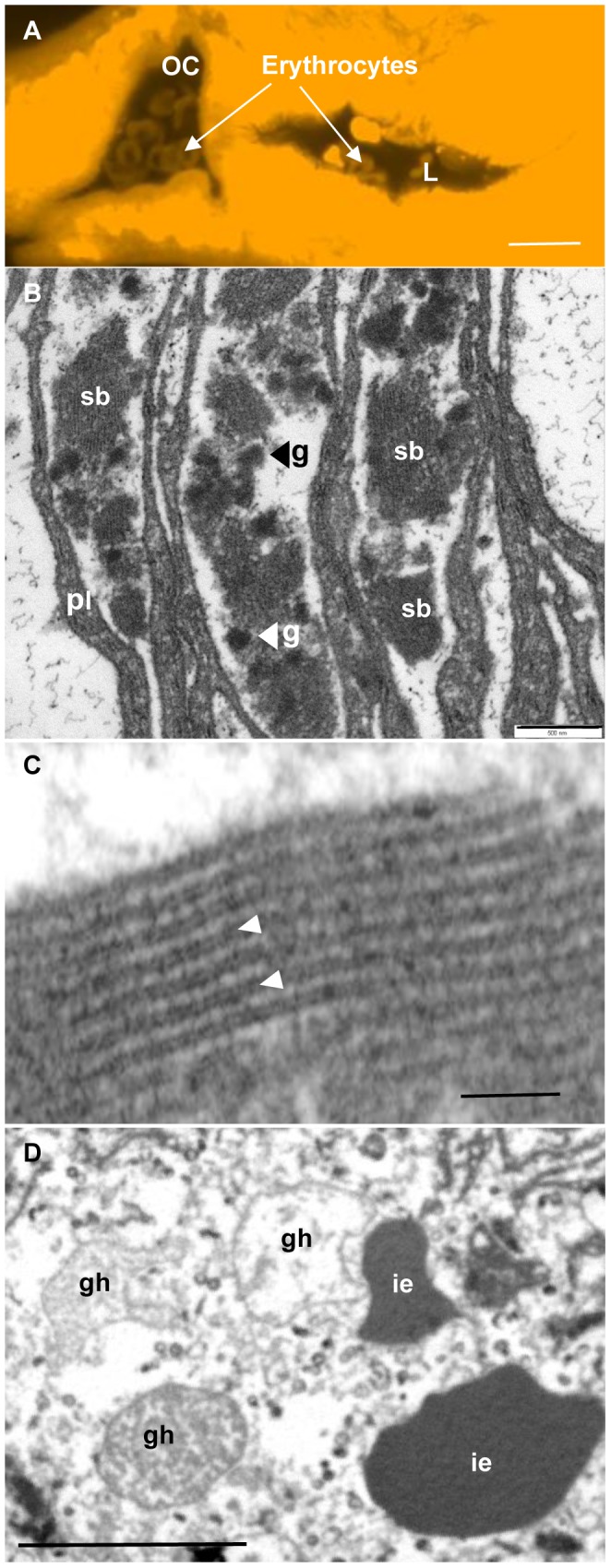
Erythrocyte processing in *S. mansoni*. (A) Confocal image of a feeding female worm fixed in vitro and stained with Langeron's carmine. Intact erythrocytes are visible within the oral cavity (OC) and the lumen (L) of the anterior esophagus. The erythrocytes can only be seen when the laser intensity is greatly amplified as they do not react well with the stain. (B) Material lying between esophageal plates (pl) of a male worm fixed in vivo, which typically has two distinct forms, small very electron dense granules (g) and looser striated bodies (sb). (C) High magnification TEM of the inter-plate aggregates showing the alternating light and dark striated material; the dark striations comprise two closely apposed layers (arrowed). (D) TEM of the posterior esophageal lumen from a male worm fixed in vivo. Three ghosts (gh) are present, showing different stages of haemoglobin leakage, plus two intact erythrocytes (ie). Scale bars: A, 10 µm; B, 500 nm; C, 100 nm; D, 5 µm.

In spite of numerous attempts, using a variety of imaging techniques, it proved difficult to ‘capture’ the moment of erythrocyte uncoating in the esophageal lumen. When worms were fixed whilst being observed feeding in vitro, intact erythrocytes could be imaged in the oral cavity and anterior esophageal bulge (Figure 6A) of both males and females. In contrast, they were not observed in the posterior esophagus or the transverse gut of the same worms. Measurement of the spaces between the plates in the posterior esophagus in TEMs revealed that they were too narrow to accommodate erythrocytes. Nevertheless, granular material was typically present in two distinct forms (Figure 6B), spherical dense bodies (160–170 nm diameter) and larger irregular shaped masses. The spherical bodies were distinct from the haematin pigment granules abundant in the gut (Figure S2C) and we initially thought that the irregular masses were the remnants of erythrocyte stroma. However, on closer inspection at high magnification, they had a striated appearance (Figure 6C), with the alternating black and white striations sufficiently well organised to suggest a quasi-molecular structure (dimensions in Table S2). Whilst the double black line was of identical width in inter-plate aggregates and the crystalloid vesicle, the white layer was twice the thickness of the electron-lucent layer in the vesicle (Table S2). Thus, the repeating unit in the aggregates was about 40% greater than that in the vesicles.

Although TEMs revealed much cellular debris in the lumen of the posterior esophagus, including whorls of membrane, obvious erythrocytes were scarce. However, in a single male worm, fixed while feeding in vitro, we observed a mixture of intact erythrocytes and clearly recognisable ghosts in different stages of haemoglobin leakage (Figure 6D). All were located close to the tips of esophageal plates. These erythrocytes and ghosts were surrounded by amorphous material, similar in appearance to the residual contents of the ghosts, presumably representing released haemoglobin. The lack of haemoglobin aggregates in the lumen of the posterior esophagus contrasts with the presence of small spheres of haemoglobin in the neighbouring transverse gut, closely adherent to the gastrodermal epithelium (Figure S2C).

### Specific genes are expressed in the esophageal gland

**Figure 7:**
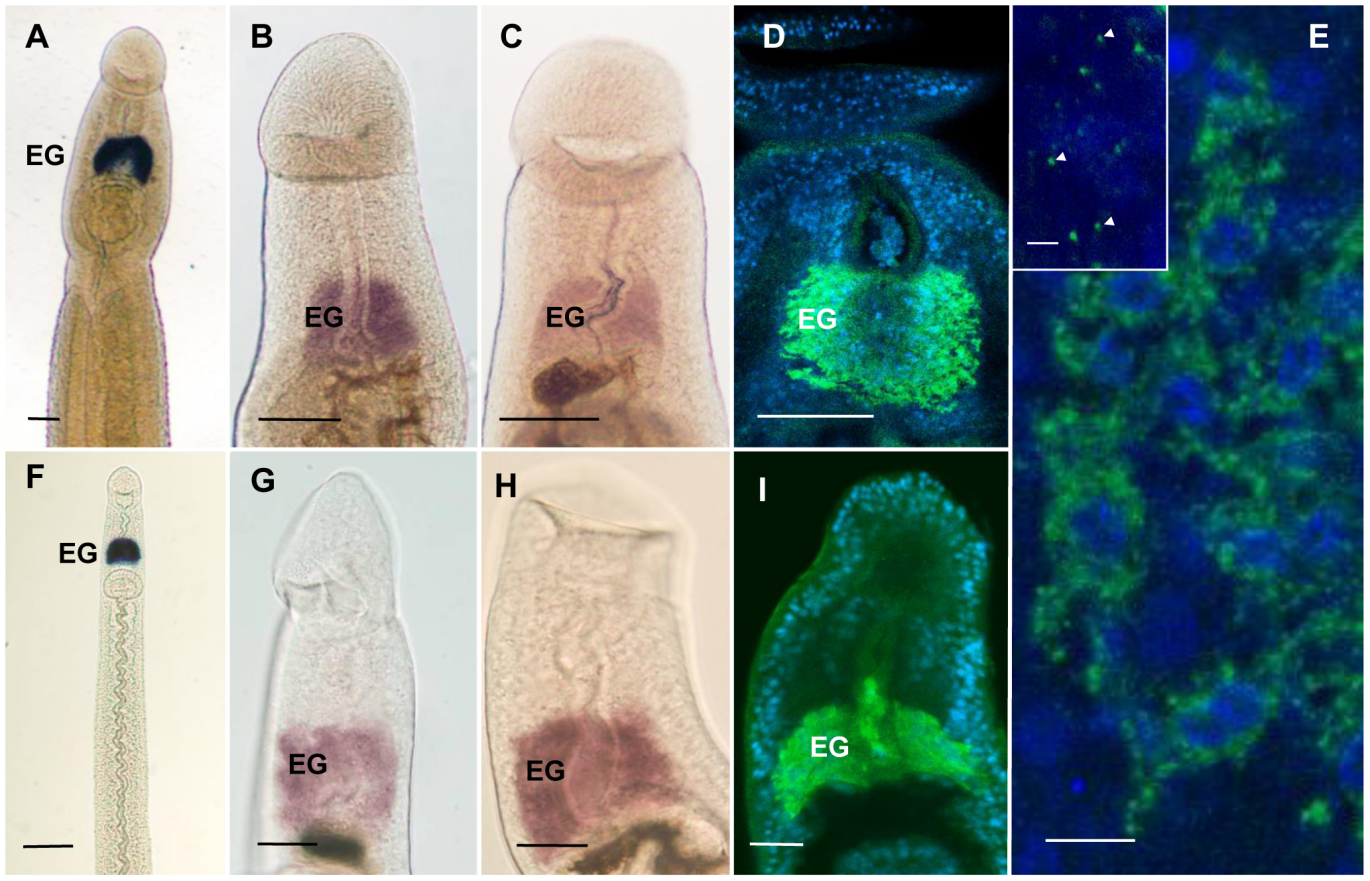
Localisation of transcripts and proteins in the esophageal gland of whole permeabilised adult worms. A–D, males; F–I, females. (A and F) Low magnification images of SmMEG-4.1 localization revealed by WISH, to show the absolute specificity of gene expression in the esophageal gland (EG). (B and G) SmMEG-14 expression solely in the esophageal gland; (C and H) SmMEG-4.2 is expressed more strongly in the female than the male esophageal gland. (D and I) Localization of *S. japonicum* MEG-4.1 protein (green) and nuclei (blue) by immunocytochemistry, in the esophageal gland. (E) High magnification of esophageal gland cell bodies reveals the abundant sites of active SjMEG-4.1 synthesis and packaging (i.e. endoplasmic reticulum and Golgi; green). The small 0.3 µm dots (inset, arrowed) at the limit of resolution are probably individual crystalloid vesicles. Scale bars: A–D, F, 100 µm; G–I, 25 µm; E, 10 µm; Inset, 2 µm.

Data from a previous microarray study of the infection process were reanalyzed to identify those genes most highly upregulated in the 3 day schistosomulum, irrespective of functions (Table S1). Among the top 20 genes, with fold increases in expression ranging from 29 to 111 times, there were five encoding putative gastrodermal proteins. The only two known esophageal gland proteins, MEG-4.1 and VAL-7, were prominent together with a further seven genes encoding MEG proteins. We therefore selected two of the MEGs, plus the *S. japonicum* homologue of MEG-4.1 to investigate the expression patterns. As previously reported, WISH revealed that the expression of MEG-4.1 in *S. mansoni* was confined exclusively to the esophageal gland of both males and females, with equal intensity (Figure 7A and F). An identical pattern of expression was observed with MEG-14 (Figure 7B and G). However, while specific localization of MEG-4.2 was identical, male worms processed in the same solutions as females showed a much lower level of expression (Figure 7C and H). Immuno-localization of *S. japonicum* MEG-4.1, using a monospecific rat antibody confirmed that, as with *S. mansoni*, there was strong specific expression in the male and female esophageal glands (Figure 7D and I). Furthermore the gland cell bodies stained intensely, indicating that MEG-4.1 is a major product of the gland secretions (Figure 7E). Indeed the tiny green dots in these micrographs probably represent individual crystalloid vesicles trafficking towards the esophageal lining (Figure 7E, inset). The MEG protein in the esophageal lumen was strongly associated with tethered leucocytes in both males and females (Figure 5C and D). This is the first demonstration of any MEG in *S. japonicum*.

### Bioinformatic analysis of esophageal gland MEG proteins reveals their novel structure

**Figure 8:**
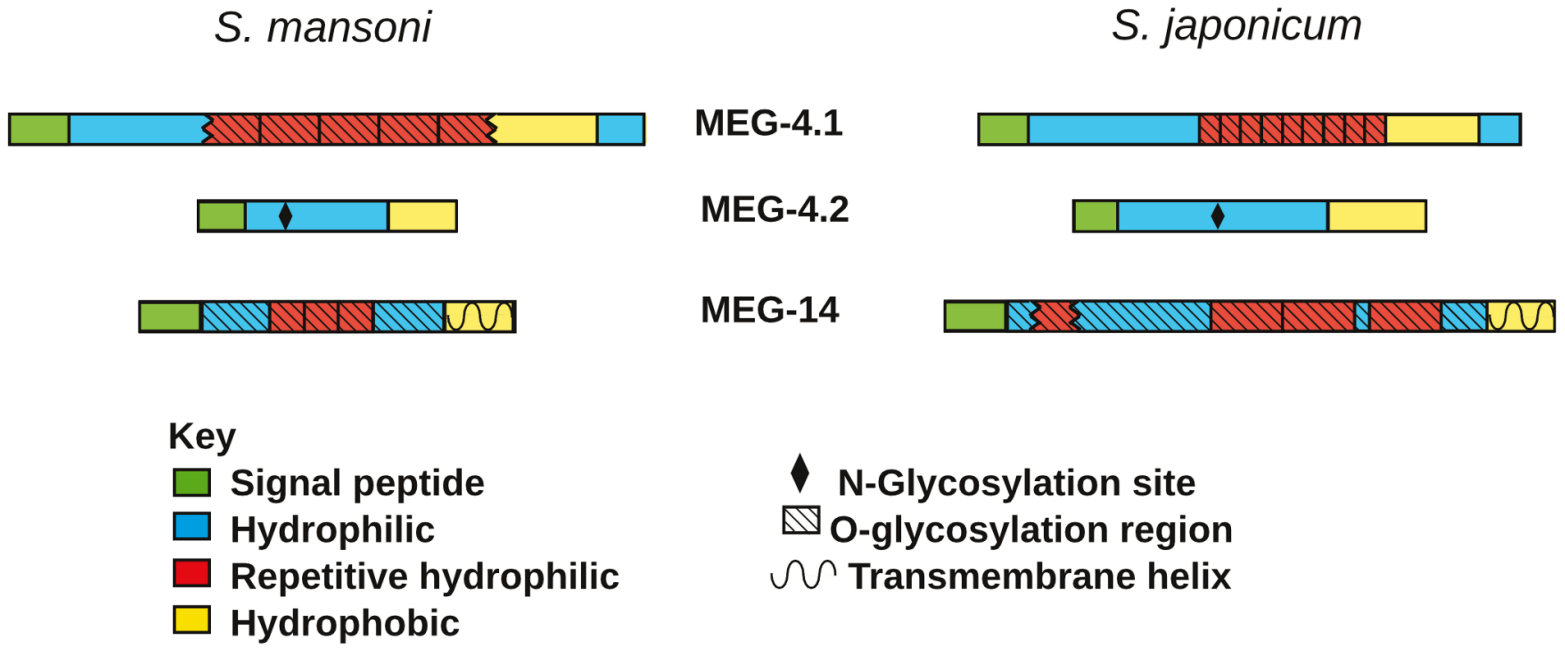
The predicted molecular properties of MEG 4.1, 4.2 and 14 proteins. The jagged edge of a motif indicates that only a partial repeat is present.

The predicted molecular properties of MEG 4.1, 4.2 and 14 proteins from *S. mansoni* and *S. japonicum* can be summarised schematically (Figure 8). The multiple repeated amino acid sequences of SmMEG-4.1 (Figure S4A, KRTTPKPTTPKQIDGTSDKTSDTHTI; three complete, two partial) were described 20 years ago [12]. A BLAST search of the NCBInr database for the nearest *S. japonicum* homologue produced CAX72670.1, with 31% identical, 43% conserved amino acids. In this instance, there were eight complete and one partial repeat of eight amino acids (Figure S4D, NNTTHPPK). Signal P predicted that both proteins possessed a signal peptide, of 24 and 21 amino acids, respectively. Parker hydrophilicity plots revealed that the central repeat regions of both proteins were very hydrophilic; in contrast, the C terminal tails were strongly hydrophobic (Figure S4B and E). As might be anticipated from the abundance of threonine and proline residues in the repeat regions, NetOGlyc predicted numerous potential O-glycosylation sites above the threshold, 44 for *S .mansoni* and 18 for *S. japonicum* (Figure S4C and F). There were no predicted N glycosylation sites.

The amino sequence of SmMEG-4.2 only showed strong homology with that of SmMEG-4.1, over a short stretch of 22 amino acids near the C terminus but no repeat regions were present (Figure 8). A BLAST search of the NCBInr database with SmMEG-4.2 revealed several sequences in *S.japonicum* with varying degrees of homology. Removing the non-overlapping amino acids from the longest *S.japonicum* sequence (likely the result of splicing out microexons) revealed a homology of 49% identical, 69% conserved amino acids, compared to *S.mansoni* (Figure S5D). As with the *S.mansoni* version, there were no repeat regions but there was a short conserved stretch of amino acids near the C terminus of SjMEG-4.2, compared to SjMEG-4.1. Signal P predicted that both Sm and SjMEG-4.2 possessed a signal peptide of 19 amino acids. Parker hydrophilicity plots of both proteins revealed a similar pattern of hydrophobic N and C termini surrounding a central hydrophilic region (Figure S5B and E). No O-glycosylation sites were predicted in the central region of either version (Figure 8, Figure S5C and F) but NetNGlyc predicted a single N-glycosylation site in the same region (Figure S5A and D).

The C terminal regions of MEGs 4.1 and 4.2 are highly conserved in all three sequenced schistosome species (*S. mansoni*, *S. japonicum* and *S. haematobium*; Figure S6). Alignment of the region in MEG-4.1 using ClustalX2 shows 36% identity, 69% conserved residues over 62 amino acids. In MEG-4.2 the agreement is 41% identity, 73% conserved residues over 59 amino acids. If all six sequences are aligned the region of homology shrinks to 22 amino acids with 27% identity and 68% conserved residues. This level of conservation within MEG-4.1 and within MEG-4.2 in the three schistosome species, and between the two MEGs over the 22 amino acid region of all sequences, suggests a shared motif executing a common function.

Although Sm and SjMEG-14 lack sequence homology to the two MEG-4 proteins, they conformed to the same general pattern of organisation. They possessed a predicted signal peptide (26 amino acids; Figure S7A and E) and a central hydrophilic region bounded by hydrophobic N and C termini (Figure S7B and F). Each central region was composed of several repeated sequences, which contained 35 and 39 O-glycosylation sites in SmMEG-14 and SjMEG-14, respectively, predicted by NetOGlyc (Figure S7C and G). The striking difference from the two MEG-4 proteins was that TMHMM predicted a single C terminal membrane-spanning region of 22 amino acids in both SmMEG-14 and SjMEG-14 (Figure S7D and H). Thus we appear to be dealing with a membrane-anchored protein with mucin-like properties.

### Western blotting reveals a gel shift of MEG-4.1, and its glycosylation

**Figure 9:**
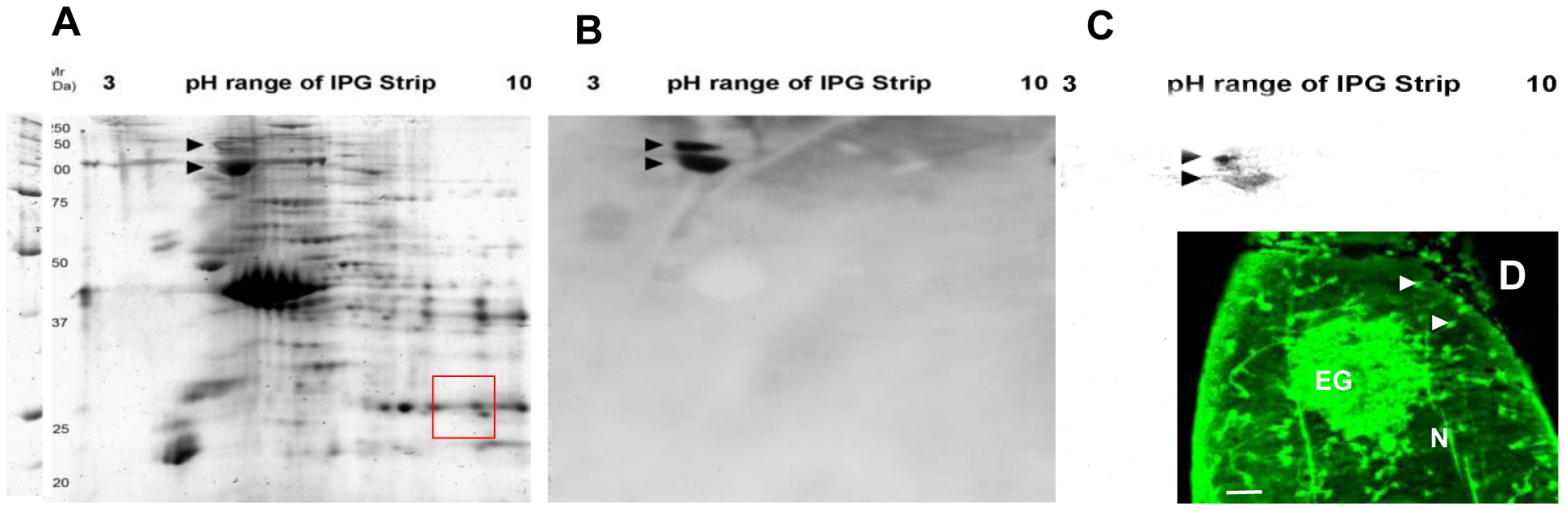
Gel shift of SmMEG-4.1 and its glycosylation. (A) 2D electrophoretic separation of head proteins after extraction. Arrows indicate actual position of Sm-MEG-4.1 protein while red square showed its predicted location. (B) Western blot of 2D gel probed with anti- SmMEG-4.1 antibody. Arrows indicate the position of the MEG protein. (C) Western blot of 2D gel probed with peanut agglutinin (PNA) reveals O-glycosylation of SmMEG-4.1 (arrowed). (D) Permeabilized whole worm reacted with FITC-labelled PNA showing reactivity of the esophageal gland (EG), nephridial canals (N) and flame cells (arrowed). Scale bar: 50 µm.

We did not detect SmMEG-4.1 on western blots of the soluble proteins extracted from whole worm homogenates and separated by 2DE. This suggested that it was present in the sample at very low concentrations so we enriched the esophageal region of male worms by excising a large number of heads. After removal of cytosolic proteins, extraction of the residual material with a mixture of chaotropic agents and detergents produced a sample suitable for 2DE (Figure 9A). The dominant constituents identified by tandem mass spectrometry were all known muscle proteins (data not shown), unsurprising considering that the head preparation contained both sucker and body wall muscles. However, when a blot of this 2D gel was probed with rat anti-SmMEG-4.1 antibody, two strong targets were detected at >100 kDa molecular weight and relatively acidic pI (Figure 9B). This is in marked contrast to the Mw and pI predicted from the amino acid sequence (27.2 kDa, 9.38, respectively). The obvious interpretation of the 70 kDa gel shift is that SmMEG-4.1 is decorated with O-glycans as predicted. Indeed, probing of a second membrane with PNA produced a reaction at exactly the same high Mw and low pI (Figure 9C), confirming the glycosylation status of SmMEG-4.1. Reaction of permeablized whole *S. japonicum* worms with FITC-labelled PNA produced intense staining of the esophageal gland. The nephridial canals and the associated flame cells also bound the PNA very strongly (Figure 9D).

### Host antibodies target antigens in the esophageal lumen

**Figure 10:**
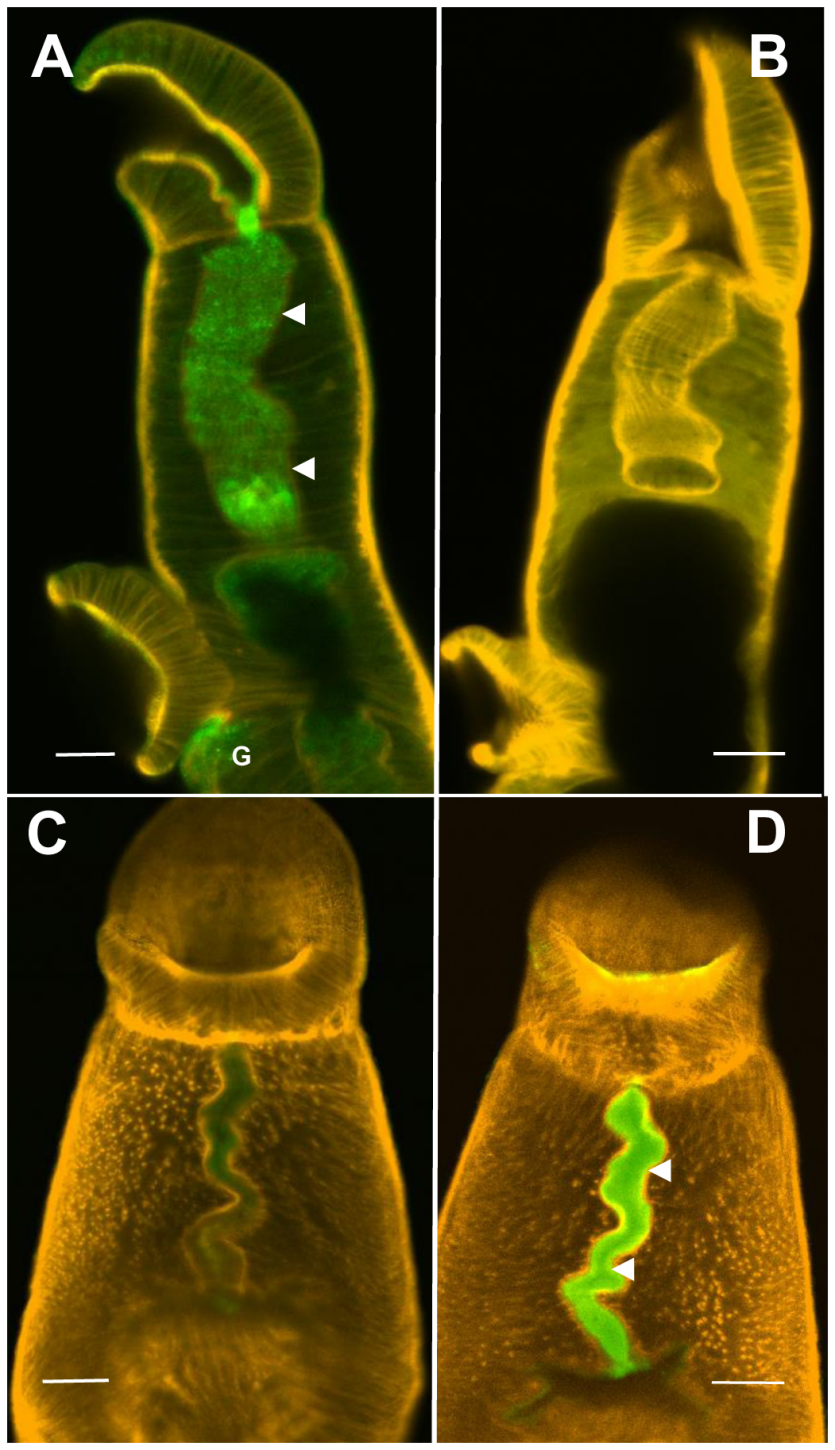
Host antibodies target antigens in the esophageal lumen of *S. mansoni*. Permeabilized female worms from mouse (A and B) and male worms from hamster (C and D) incubated with fluorescent-labelled antibodies (green) and counterstained with phalloidin (orange) to highlight the musculature. (A) Test with anti-mouse IgG. (B) Control with anti-hamster IgG. (C) Control with anti-mouse IgG. (D) Test with anti-hamster IgG. Strong specific antibody binding was only detected in the esophageal lumen (arrowed) of the relevant parasite, plus the gonopore region (G) of the female from mouse. Scale bars: A & B, 20 µm; C & D, 50 µm.

The reactivity of the contents of the esophageal lumen in worms ex-vivo was investigated using anti-host IgG antibodies. Permeabilized worms from mice showed strong reactivity with mouse IgG over the whole length of the esophageal lumen (Figure 10A), most intense at the oral sphincter and towards the posterior, together with a region around the gonopore. The mouse worms did not react with anti-hamster IgG (Figure 10B). The exact reverse situation was observed with hamster worms where anti-hamster IgG reacted with the esophageal lumen (Figure 10D) but anti-mouse IgG did not (Figure 10C). The intense positive staining was uniformly distributed along the whole length of the esophageal lumen.

## Discussion

The schistosome esophagus is the first point of interaction with incoming blood. Structurally, it is clearly demarcated into anterior and posterior regions, coincident with two distinct phases of blood processing. The network of micron diameter circular and longitudinal muscle fibres embedded in the structure, generate the forward peristalsis to propel blood from the oral cavity to the transverse gut. Their wider spacing, compared those of the close-packed sub-tegumental musculature, is presumably to accommodate the expansions and contractions in peristalsis. Although no sphincter was observed dividing the two regions, incoming blood accumulates in the anterior lumen before passing as a bolus to the posterior, indicating fine neurological control of the musculature. We have been unable to discover who first designated the cell bodies of the posterior esophagus as a gland but those of the anterior, although almost as numerous, have not been singled out in this way. The products of the anterior cell bodies are typical of the surface tegument but why such an intense concentration of cell bodies is required remains unclear. The most obvious function for the longitudinal corrugations of the anterior lining is to increase the interaction-surface area, but they may also orientate the incoming cells for later processing.

In contrast, the cell bodies of the posterior esophagus manufacture the unique crystalloid vesicles that replace the multilaminate vesicles of the anterior, so no membranocalyx is present. Their finely crystalline structure was first remarked upon by Morris & Threadgold [1] and described in detail by Bogitsh and Carter [3], who interpreted the contents as parallel layers of membrane. The vesicles are 0.26×0.21 µm in size (not the 4.0×2.0 µm given by Bogitsh and Carter) and we differ in interpreting the contents as quasi-molecular structures of highly ordered proteins/glycoproteins that have self-assembled after packaging in the Golgi apparatus of the gland cell bodies. The salient feature of the posterior esophageal syncytium is its very large surface area. This is provided by the combination of the thin plate-like extensions of the luminal membrane and the very elongate invaginations of the basal membrane that penetrate almost the whole length of each plate. (The plates are not analogous to the much shorter surface extensions of the gastrodermis; [9]). Ostensibly, this increased surface area would enhance interactions with incoming cells but the spaces between plates are too narrow to admit erythrocytes or leucocytes, and quite possibly platelets, leaving only plasma proteins able to enter. The basal invaginations are typical of epithelial cells such as those of the proximal and distal tubules of the mammalian kidney [19], where ion transporters are located. The Na-K-Cl cotransporter is one such protein in the basolateral membranes of secretory epithelia in many species [20]. This raises the possibility that the enormous surface area is an adaptation to generate massive ion fluxes into or out of the posterior esophagus lumen, and this in turn might be linked to erythrocyte lysis.

The location of crystalloid vesicles in the base of the syncytium and a short distance up the plates indicates that secretion of contents occurs only in this region. The contained parallel array of material is released intact to the lumen, at specific docking sites, by fusion of the vesicle membrane with the surface plasma membrane [3]. Thereafter the arrays do not dissipate, as would be expected for normal secretory vesicle contents, but persist intact, as attested in several studies [2], [3], [5], [21]. Indeed our observations show that they cluster into larger aggregates, thus confirming the self-affinity of their molecular constituents. However, the 40% greater repeating unit in the aggregates, compared to the vesicles, indicates that expansion of the electron lucent layers occurs after release. These observations provide another potential function of the plates, namely to sequester the crystalloid aggregates, allowing them both to interact more efficiently with the incoming blood and to prevent their wash-out (and waste) when the worm vomits. This hypothesis envisages the longitudinal plates of the posterior esophagus serving as a slow release reservoir for gland products that must interact instantaneously when blood is ingested. In the context of wash-out by vomit, we did not observe reverse peristalsis of the musculature. Rather, vomiting was characterised by a thin stream of material up the esophageal lumen, powered by contractions in the gut. Such a configuration of the esophagus would minimize the loss of gland secretions.

A remarkable feature of the posterior esophagus of both *S. mansoni* and *S. japonicum*, in males and females, is the extended cluster of leucocytes occupying the centre of the lumen. Previously, such cells were only mentioned in passing in two TEM studies [1], . The stationary position of the cells in consecutive video frames and their battered appearance in TEMs confirms that they are tethered in this location. How this is achieved remains unclear but the inference must be that tethering benefits the worm by minimising leucocyte transit to the gut lumen. This would avoid production of an oxidative burst in the gut in proximity to the reproductive organ with their rapidly dividing cells but conversely would require highly active anti-oxidant systems in a very concentrated region of the esophagus. The tethering is leucocyte-specific as blood can be seen in movies, flowing round the sequestered mass of nucleated cells. That many trapped leucocytes are damaged implies secretion of a ‘toxic agent’ from the esophageal gland.

The number of leucocytes in the posterior esophageal lumen of male *S.mansoni* is three times that of females, roughly in proportion to their respective size. The investigation by Lawrence of blood feeding in vivo, estimated that males and females ingested 39,000 and 330,000 erythrocytes per hour, respectively [8]. Although the sole study, its conclusions are reinforced by the disparities in the amounts of haemozoin (the haemoglobin breakdown pigment) in males and females [22]. From blood feeding data we can estimate the number of leucocytes ingested per hour as 54.6 for males and 462 for females. Given the average number of leucocytes observed in a male (28), the ‘duration of stay’ in the lumen equates to 31 minutes. The battered state of tethered leucocytes, observed by TEM, indicates that they are destroyed in situ. The 31-minute ‘duration of stay’ is too short for apoptosis, which is measured in hours [23] but more consistent with necrosis, induced either directly by parasite-generated toxins or indirectly via the leucocyte's own necrosis pathways, e.g. by activation of RIP-1 (receptor interacting protein) kinase [24]. If the much greater ingestion of blood by females is analysed in this way, the duration of leucocyte stay (10.4 observed/462 predicted) would be only 1.5 minutes, a 21 fold discrepancy in timing between males and females. It seems unlikely that females could destroy leucocytes so much faster. It is possible that they ingest fewer leucocytes by selectively excluding them due to their smaller mouths or that many pass into the gut lumen. However, leucocytes observed in that location are severely damaged. Conversely, following tethering and damage, leucocytes could be expelled with vomitus since females feed (and vomit) at 8 to 9 times the rate of males [8].

We have already shown, in earlier feeding experiments [9], that the lipophilic PKH2 compound transfers from the erythrocyte membrane to the posterior esophageal lining. Furthermore, intact erythrocytes can be visualized in the anterior esophageal compartment by confocal microscopy (this study) and TEM [7], but not in the transverse gut where the haemoglobin is aggregated into micron size blobs [9]. Indeed, Spence and Silk in their extensive ultrastructural study, recorded that they did not observe intact red blood cells or ghosts in any TEM section [21]. We thus infer that haemolysis occurs in the posterior esophagus but the extreme scarcity of erythrocyte ghosts implies that it is a rapid process. From video analysis, we estimated that erythrocytes take only 4 to 5 seconds to pass through the posterior esophagus during which haemoglobin leaks out. Thus the fate of erythrocytes differs markedly from that of leucocytes, not just in the absence of tethering but also in the manner of destruction, potentially due to their greater fragility and susceptibility to osmotic/ionic changes. One outstanding question to be resolved is that if haemoglobin is released into solution in the esophageal lumen, how does it aggregate to form blobs in the gut? Is it purely physical as a result of pH change (pH of the gut is acidic, ∼4.8, [22]; <6.2 and inferred 4.9, [9]) or chemical interaction? The potential value to the worm of anchoring blobs of undigested haemoglobin to the gastrodermal lamellae is that they, and the nutrients they represent, will be retained when haemozoin pigment is regurgitated in vomitus.

SmMEG-4.1 was the first protein to be localized in the esophageal gland [10]. It was also one of the 20 most highly up-regulated genes in the day 3 schistosomulum, as was VAL-7, recently also localized to the gland [13]. We have now demonstrated that two further genes in the top 20 list, SmMEG-14 and SmMEG-4.2, are also expressed in the gland. The additional presence of five putative gastrodermal genes in the top 20 list indicates that this tissue, along with the esophageal gland, is undergoing differentiation ahead of worm arrival in the portal vein and the start of blood feeding. (The expression sites of MEG-8.1, 9, 11 and 12 also highlighted by the array, are currently being investigated.) It is tempting to conclude that the four proteins identified so far are principal constituents of the secretory crystalloid vesicles. The predicted molecular weight of the longest isoform of MEG-4.1, based on amino acid composition, is 27.4 kDa, but the observed molecular weight is between 110 and 120 kDa, due at least in part to the decorating O-glycans. Depending on configuration, its dimensions would range from 9.6 nm (sphere) to 18 nm (rod) [25]. Thus the repeating unit of the crystalloid vesicles and inter-plate aggregates could accommodate two such protein molecules lined up in apposition, with their peptide backbones forming the dark line and the decorating glycans filling the space between. Indeed, the 40% greater size of the repeating unit in the inter-plate aggregates, compared to the vesicles, could be due to hydration of the O-glycan chains after secretion.

Western blotting and lectin staining of protein extracts from worm heads confirmed the bioinformatic prediction that MEG-4.1 was heavily O-glycosylated. It also revealed that at least two isoforms were present, potentially the product of alternative splicing of microexons or differential glycosylation. The strong PNA lectin staining of the esophageal gland, recently reported for *S. mansoni* [14] was also confirmed here in *S. japonicum*. We suggest the presence of large amounts of O-glycans on the native MEG-4.1 is likely to make it a very sticky macromolecule, prone to adhere to surfaces. This is supported by its immunodetection not just in the lumen of the gland but also up the entire esophagus, way beyond its secretion zone [11]. MEG-14 is also predicted to be O-glycosylated but the membrane anchor and predicted extent of glycosylation suggest it is mucin-like, potentially providing a surface coat for the entire lumen of the posterior esophagus. This idea is supported by early observation of neutral mucosubstances (revealed by PATCO staining) at the surface of the esophageal lining in a related parasite (*Schistosomatium douthitti*; [5]). In contrast, MEG-4.2 is predicted to be secreted but not O-glycosylated, so more likely to exert its function in solution. The most important feature common to MEG-4.1 and MEG-4.2 is a motif conserved throughout schistosome evolution. Examination of the consensus sequence using Jpred (http://www.compbio.dundee.ac.uk/www-jpred/) predicts an alpha helix configuration. Furthermore, the detection of MEG-4.1 associated with leucocytes in the esophageal lumen, suggests that it targets these cells, e.g. by binding to a pan-leucocyte marker such as CD45. The function of the secreted VAL-7 is more problematic since its Sperm Coating Protein (SCP) domain is widespread in proteins from many organisms, yet no clear molecular function has been defined. There are at least 28 members in the SmVAL superfamily [26], [27], but SmVAL-7 appears to be the most distantly related of all the Group 1 (secreted) VALs [27]. Extended similarity group analysis [28] suggests ion channel inhibitor activity as the molecular function for VAL-7, with a probability of 80% for *S. mansoni* and 65% for *S. japonicum*. Such a property could affect the viability of incoming host cells, a hypothesis worth testing.

Detection of host IgG binding in the esophageal lumen raises the possibility that the esophageal proteins might serve as targets of the host immune response. Clearly, in the permissive hamster and mouse worms we examined, this binding is not detrimental to parasite survival. It would be instructive to repeat these experiments with worms from non-permissive/self-curing hosts, such as the laboratory rat and rhesus macaque [29], [30]. Another important task is to identify more potential targets secreted by the esophageal gland beyond the four we already know. Potentially, the novel proteins of the esophageal gland make attractive vaccine candidates, not least because they are secreted into a confined space to mediate key aspects of the feeding process. In addition, vaccine-induced antibodies that targeted such proteins would not face the hostile acid proteolytic environment that surrounds targets in the gut lumen or on the gastrodermis.

## Supporting Information

Figure S1 **Cellular morphology of the esophageal gland cell bodies and granule traffic revealed by TEM.** (A) Esophageal gland of *S.mansoni* showing several cell bodies in section with large amounts of rough endoplasmic reticulum (rER) indicating intense protein synthesis for export. (B) The same region in *S.japonicum* at lower magnification showing the very elongated cell bodies that convey crystalloid vesicles towards the esophageal lining; in both (A) and (B), the nuclei contain very prominent nucleoli (N). (C) An assemblage of crystalloid vesicles (white arrows) in proximity to a Golgi body (starred) in an esophageal gland cell of *S. japonicum*. Microtubles (black arrows) are present in the vicinity of granules, suggesting that their transport occurs using kinesin motors. (D) Crystalloid vesicle (CV) traffic from the cell bodies, through the muscle layers (M), to the syncytium, via narrow cytoplasmic connections (CC) containing microtubules (arrowed). (E) Esophageal plates showing the origin of the twin central lines (arrowed) as invaginations (IN) of the basal plasma membrane. Crystalloid vesicle (CV) and discoid body (DB), products of the gland cells, are visible in the plate cytoplasm. Scale bars: A & B, 2 µm; C, 500 nm; D, 1 µm; E, 500 nm.
(TIF)

Figure S2 **Worm vomiting and gut function in** ***S. mansoni*** **.** (A) A continuous stream of pigment is passing from the transverse gut (TG) up the open lumen (arrowed) of both posterior and anterior esophagus. (B) The vomit exits the oral cavity (OC) as a stream of fine particulate material comprising hemozoin pigment (arrowed). (C) TEM of the transverse gut of a male worm showing the micron sized blobs (B) of aggregated haemoglobin intimately associated with the lamellar extensions (LE) of the gastrodermis. Highly dense masses of hemozoin pigment (H) are abundant throughout the lumen. Scale bars: A, 100 µm; B, 50 µm; C, 2 µm.
(TIF)

Figure S3 **Overview of leucocytes detained in the posterior esophageal lumen and their remnants in the gut of** ***S.mansoni*** **.** (A) A single frame from a feeding movie, representative of consecutive images, showing the stationary plug (P) of cells within the lumen of the esophageal gland (outlined). (B) TEM of posterior esophagus in transerse orientation showing its elliptical shape, with a mass of host neutrophils (N) and mononuclear cells (M) in the lumen center and other cell debris (D) interacting with the plate (PL) tips. (C, D) TEM of the transverse gut showing damaged mononuclear leucocytes (M) adjacent to the gastrodermal epithelium (GE) and its lamellar extensions (LE). Scale bars: A, 100 µm; B, 10 µm; C & D, 2 µm.
(TIFF)

Figure S4 **Bioinformatic analysis of MEG-4.1.** (A) Amino acid sequence, (B) Parker hydrophilicity plot and (C) O-glycosylation sites predicted by NetOGlyc, of *S. mansoni* (SmMEG-4.1). (D) Amino acid sequence, (E) Parker hydrophilicity plot and (F) O-glycosylation sites predicted by NetOGlyc, of *S. japonicum* (SjMEG-4.1). For A and D, the signal peptide is shown in blue, repeats are indicated in bold separated by a vertical line and the immunogenic synthetic peptide is underscored.
(TIF)

Figure S5 **Bioinformatic analysis of MEG-4.2.** (A) Amino acid sequence, (B) Parker hydrophilicity plot and (C) O-glycosylation sites predicted by NetOGlyc, of *S. mansoni* (SmMEG-4.2). (D) Amino acid sequence, (E) Parker hydrophilicity plot and (F) O-glycosylation sites predicted by NetOGlyc, of *S. japonicum* (SjMEG-4.2). For A and D, the signal peptide is shown in blue and the one N glycosylation site predicted by NetNGlyc is shown in red.
(TIF)

Figure S6 **Clustal analysis of the C-terminal region in MEG-4 genes from three species.** (A) MEG-4.1, (B) MEG-4.2, (C) The homologous region in all six sequences (underscored in A, B and C).
(TIF)

Figure S7 **Bioinformatic analysis of MEG-14.** (A) Amino acid sequence, (B) Parker hydrophilicity plot, (C) O-glycosylation sites predicted by NetOGlyc and (D) transmembrane region predicted by TMHMM , of *S. mansoni* (SmMEG-14). (E) Amino acid sequence, (F) Parker hydrophilicity plot, (G) O-glycosylation sites predicted by NetOGlyc and (H) transmembrane region predicted by TMHMM, of *S. japonicum* (SjMEG-14). For A and E, the signal peptide is shown in blue and repeats are indicated in bold separated by a vertical line. Single amino acid substitutions in the repeats of SjMEG-14 are shown in red.
(TIF)

Movie S1 **Cell masses in the anterior and posterior esophageal region of** ***S. japonicum*** **.** A Z-stack of the esophageal region in a male worm taken on the Zeiss 780 Multiphoton microscope allows estimation of the total cell number by counting of nuclei (light blue).
(MOV)

Movie S2 **Sweeping action of the oral sucker gathering erythrocytes for ingestion.** The oral sucker of a male worm sweeps erythrocytes into the oral cavity in a rapid grabbing motion. The anterior esophageal bulge, indicated by a white circle, slowly inflates as the erythrocytes enter. Note that the worms can only be viewed for filming in a low concentration of erythrocytes and the bulge presumable fills more quickly in whole blood. A plug of host leucocytes is visible in the posterior bulge, with cells stationary in consecutive frames.
(MOV)

Movie S3 **Transit of ingested blood in esophagus.** The dark bulge of blood cells passes from the anterior to the posterior esophagus.
(MP4)

Movie S4 **Passage of ingested blood around the plug of material in the posterior esophagus.** High magnification image of blood passing through the posterior esophagus of a male worm (from bottom left to top right) around a plug of leucocytes (white material, labelled) in the posterior esophageal lumen. As the dark material passes around the plug, it potentially interacts with the esophageal lining. Erythrocytes ghosts are visible at some time points. Note that because the worm is active, the plug goes in and out of focus.
(MOV)

Table S1 ***S. mansoni*** **Infection array data arranged by fold change in expression between germ ball and day 3 schistosomulum.**
(XLS)

Table S2 **Quantitative aspects of the esophageal region morphology.**
(XLS)

Table S3 **Volumes and cell capacity of esophageal luminal compartments (bulges).**
(XLS)
