# Supplementary figures and images for: The Schistosome Oesophageal Gland: Initiator of Blood Processing
Source: PLoS Negl Trop Dis. 2013 Jul 25;7(7):e2337. doi: 10.1371/journal.pntd.0002337 (PMC3723592; doi:10.1371/journal.pntd.0002337)

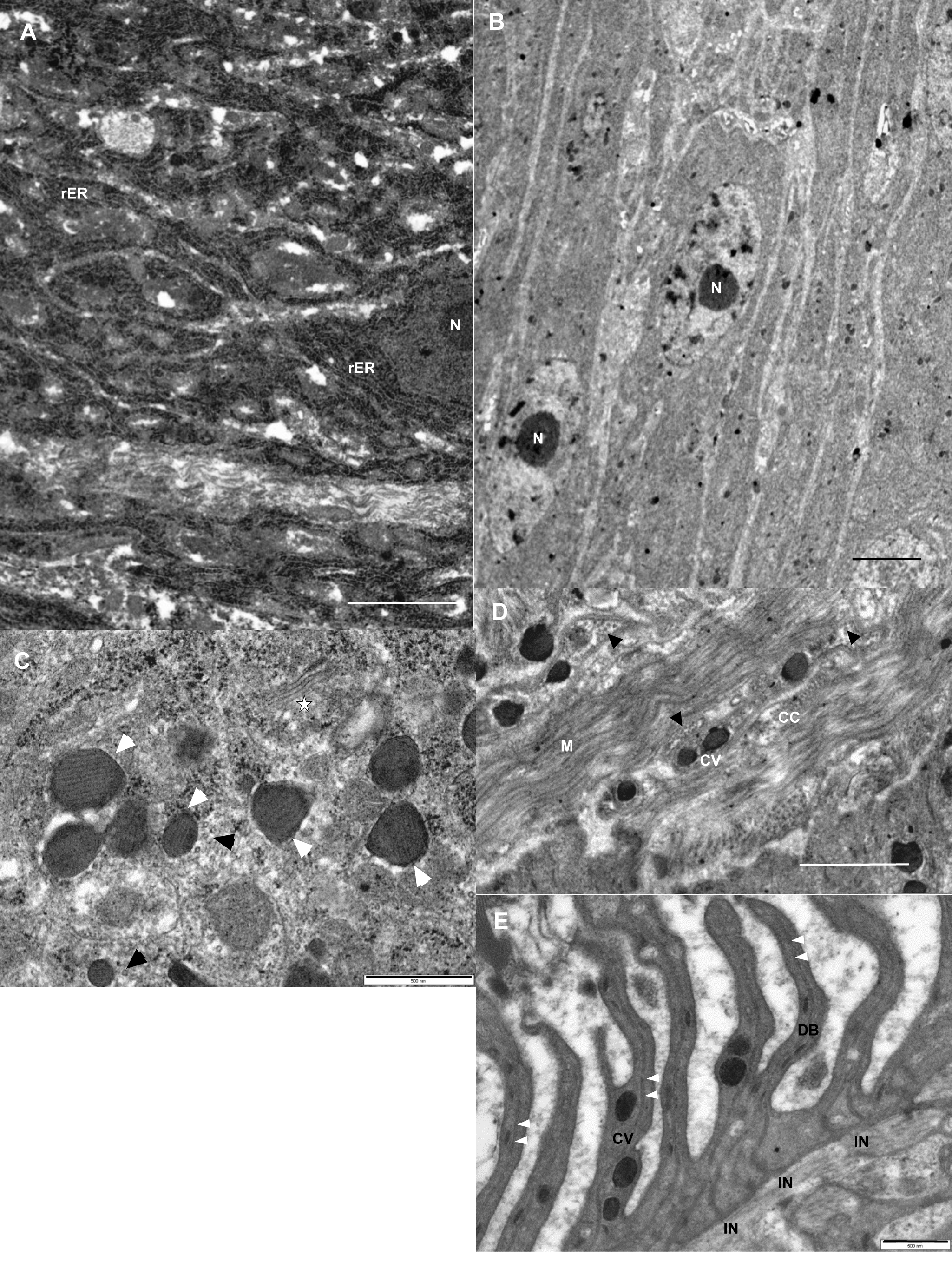

Supplement: Figure S1 — Cellular morphology of the esophageal gland cell bodies and granule traffic revealed by TEM. (A) Esophageal gland of S.mansoni showing several cell bodies in section with large amounts of rough endoplasmic reticulum (rER) indicating intense protein synthesis for export. (B) The same region in S.japonicum at lower magnification showing the very elongated cell bodies that convey crystalloid vesicles towards the esophageal lining; in both (A) and (B), the nuclei contain very prominent nucleoli (N). (C) An assemblage of crystalloid vesicles (white arrows) in proximity to a Golgi body (starred) in an esophageal gland cell of S. japonicum. Microtubles (black arrows) are present in the vicinity of granules, suggesting that their transport occurs using kinesin motors. (D) Crystalloid vesicle (CV) traffic from the cell bodies, through the muscle layers (M), to the syncytium, via narrow cytoplasmic connections (CC) containing microtubules (arrowed). (E) Esophageal plates showing the origin of the twin central lines (arrowed) as invaginations (IN) of the basal plasma membrane. Crystalloid vesicle (CV) and discoid body (DB), products of the gland cells, are visible in the plate cytoplasm. Scale bars: A & B, 2 µm; C, 500 nm; D, 1 µm; E, 500 nm. (TIF) [file pntd.0002337.s001.tif]

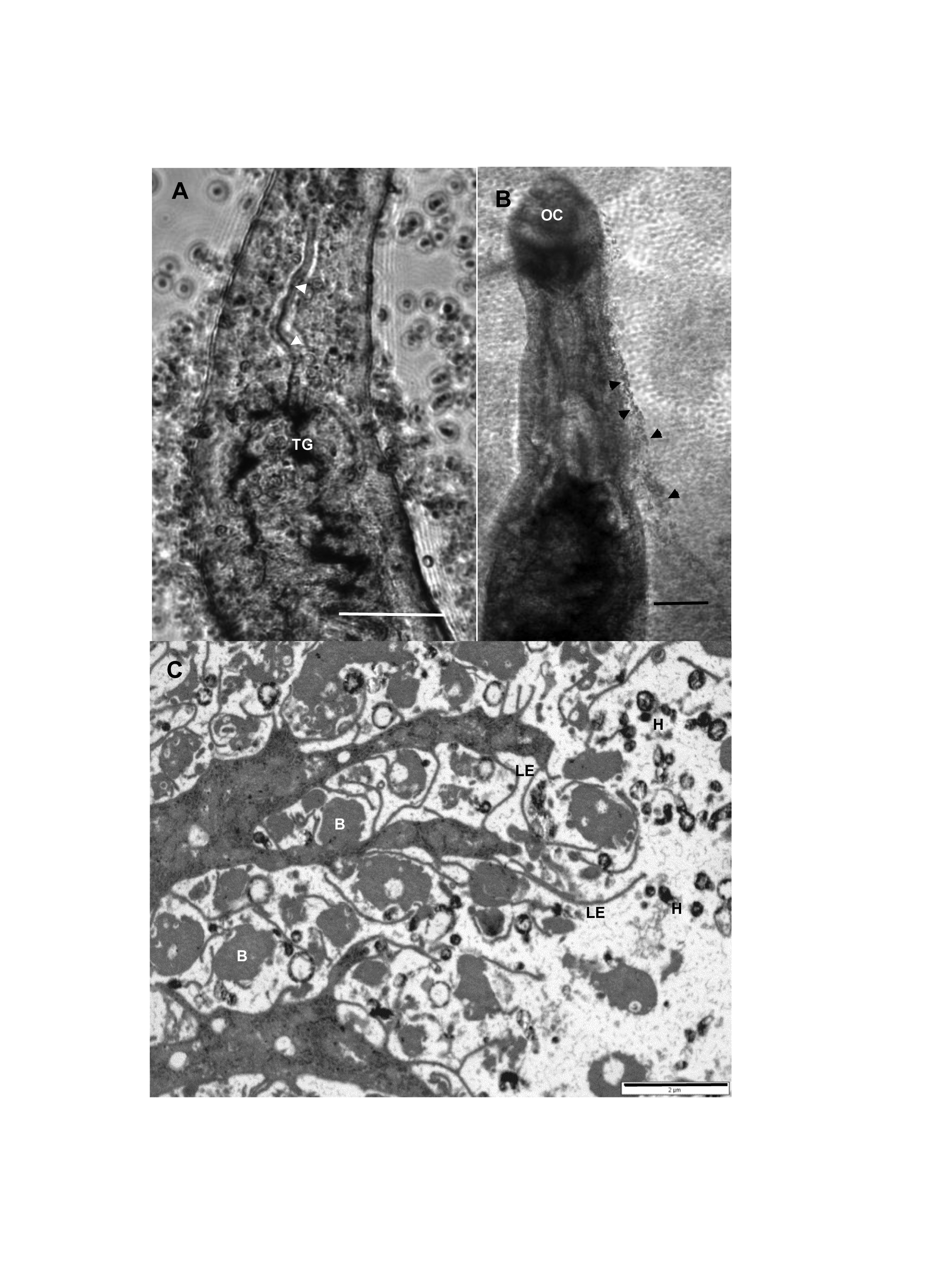

Supplement: Figure S2 — Worm vomiting and gut function in S. mansoni . (A) A continuous stream of pigment is passing from the transverse gut (TG) up the open lumen (arrowed) of both posterior and anterior esophagus. (B) The vomit exits the oral cavity (OC) as a stream of fine particulate material comprising hemozoin pigment (arrowed). (C) TEM of the transverse gut of a male worm showing the micron sized blobs (B) of aggregated haemoglobin intimately associated with the lamellar extensions (LE) of the gastrodermis. Highly dense masses of hemozoin pigment (H) are abundant throughout the lumen. Scale bars: A, 100 µm; B, 50 µm; C, 2 µm. (TIF) [file pntd.0002337.s002.tif]

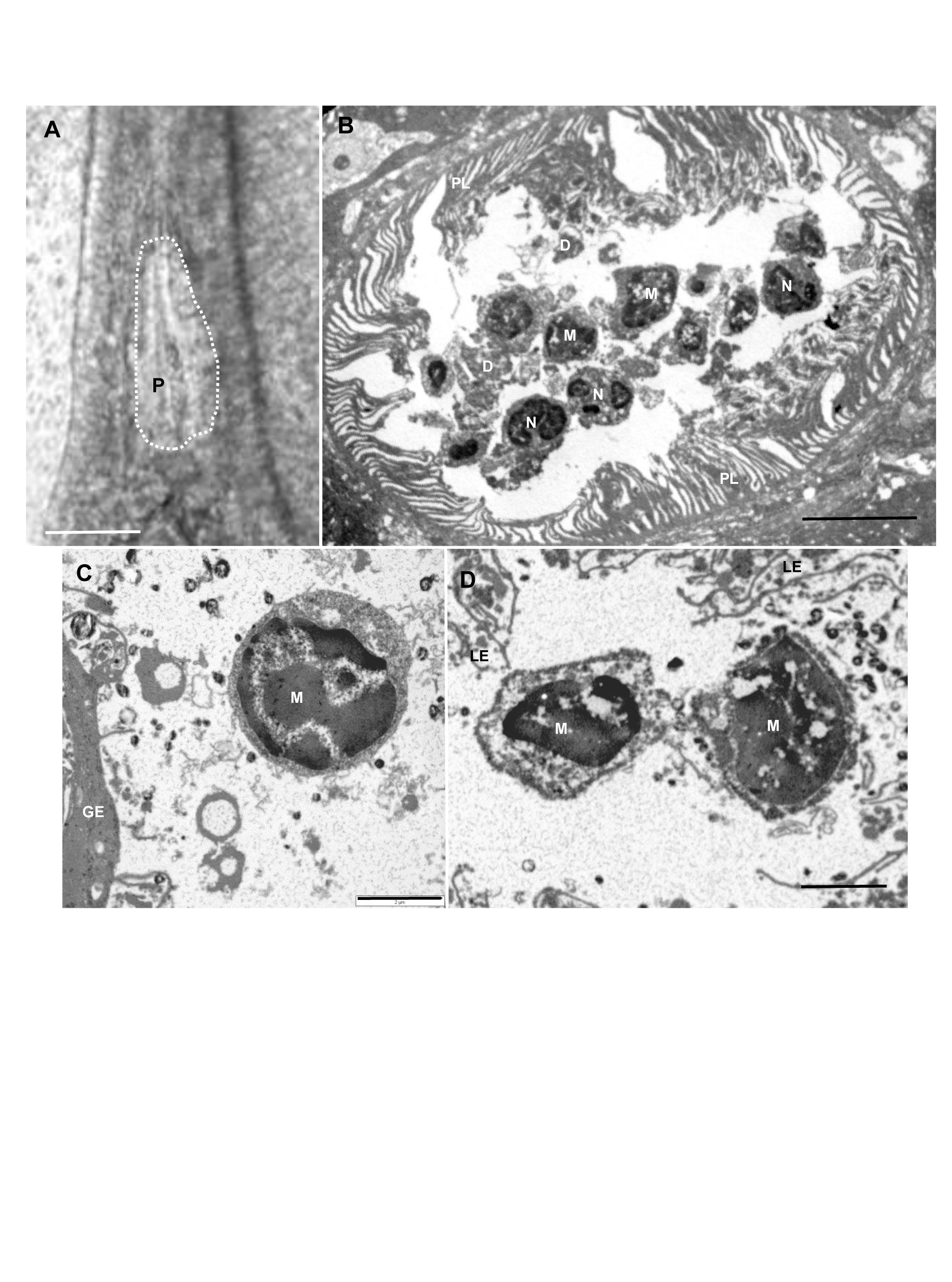

Supplement: Figure S3 — Overview of leucocytes detained in the posterior esophageal lumen and their remnants in the gut of S.mansoni . (A) A single frame from a feeding movie, representative of consecutive images, showing the stationary plug (P) of cells within the lumen of the esophageal gland (outlined). (B) TEM of posterior esophagus in transerse orientation showing its elliptical shape, with a mass of host neutrophils (N) and mononuclear cells (M) in the lumen center and other cell debris (D) interacting with the plate (PL) tips. (C, D) TEM of the transverse gut showing damaged mononuclear leucocytes (M) adjacent to the gastrodermal epithelium (GE) and its lamellar extensions (LE). Scale bars: A, 100 µm; B, 10 µm; C & D, 2 µm. (TIFF) [file pntd.0002337.s003.tif]

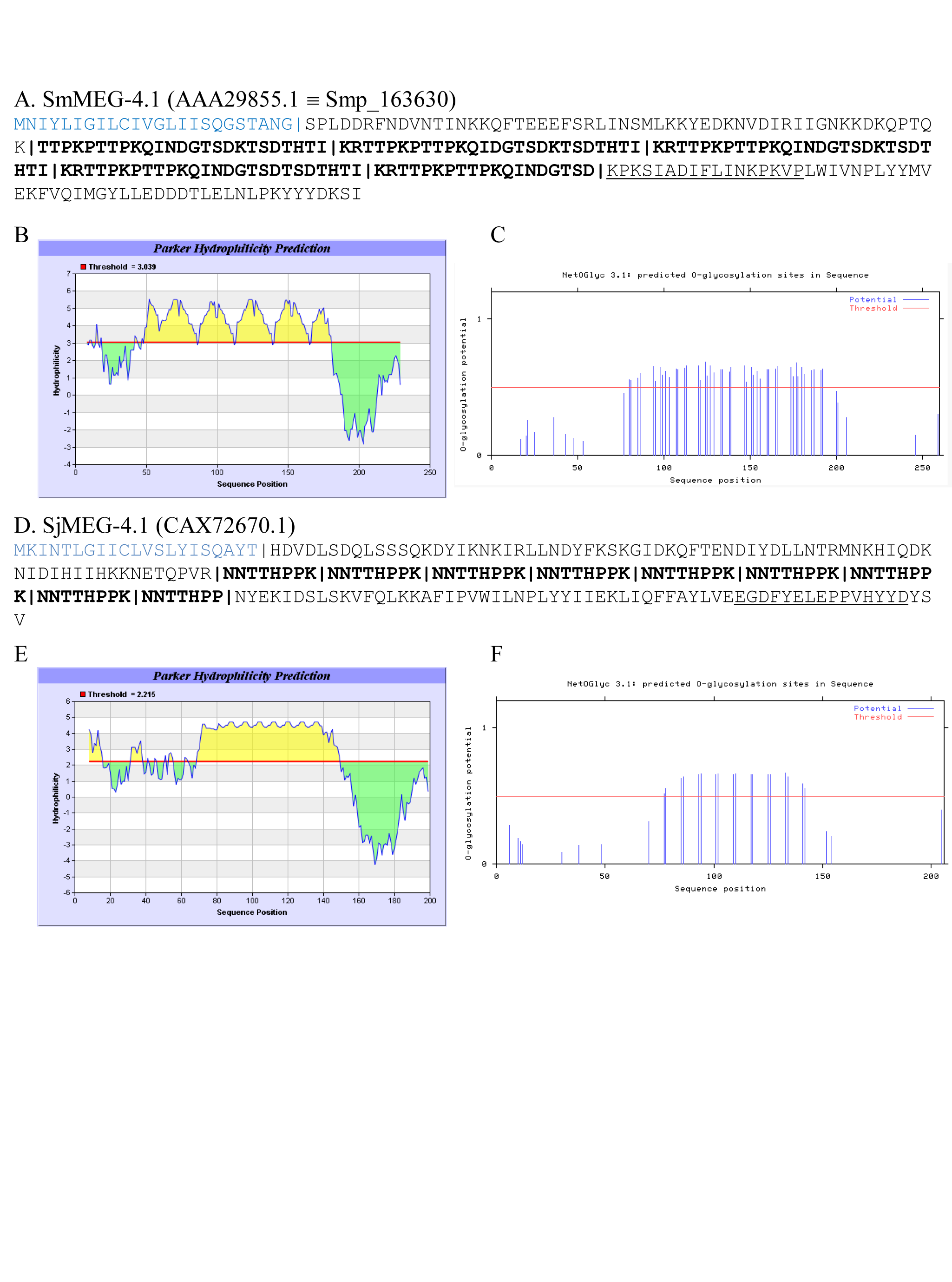

Supplement: Figure S4 — Bioinformatic analysis of MEG-4.1. (A) Amino acid sequence, (B) Parker hydrophilicity plot and (C) O-glycosylation sites predicted by NetOGlyc, of S. mansoni (SmMEG-4.1). (D) Amino acid sequence, (E) Parker hydrophilicity plot and (F) O-glycosylation sites predicted by NetOGlyc, of S. japonicum (SjMEG-4.1). For A and D, the signal peptide is shown in blue, repeats are indicated in bold separated by a vertical line and the immunogenic synthetic peptide is underscored. (TIF) [file pntd.0002337.s004.tif]

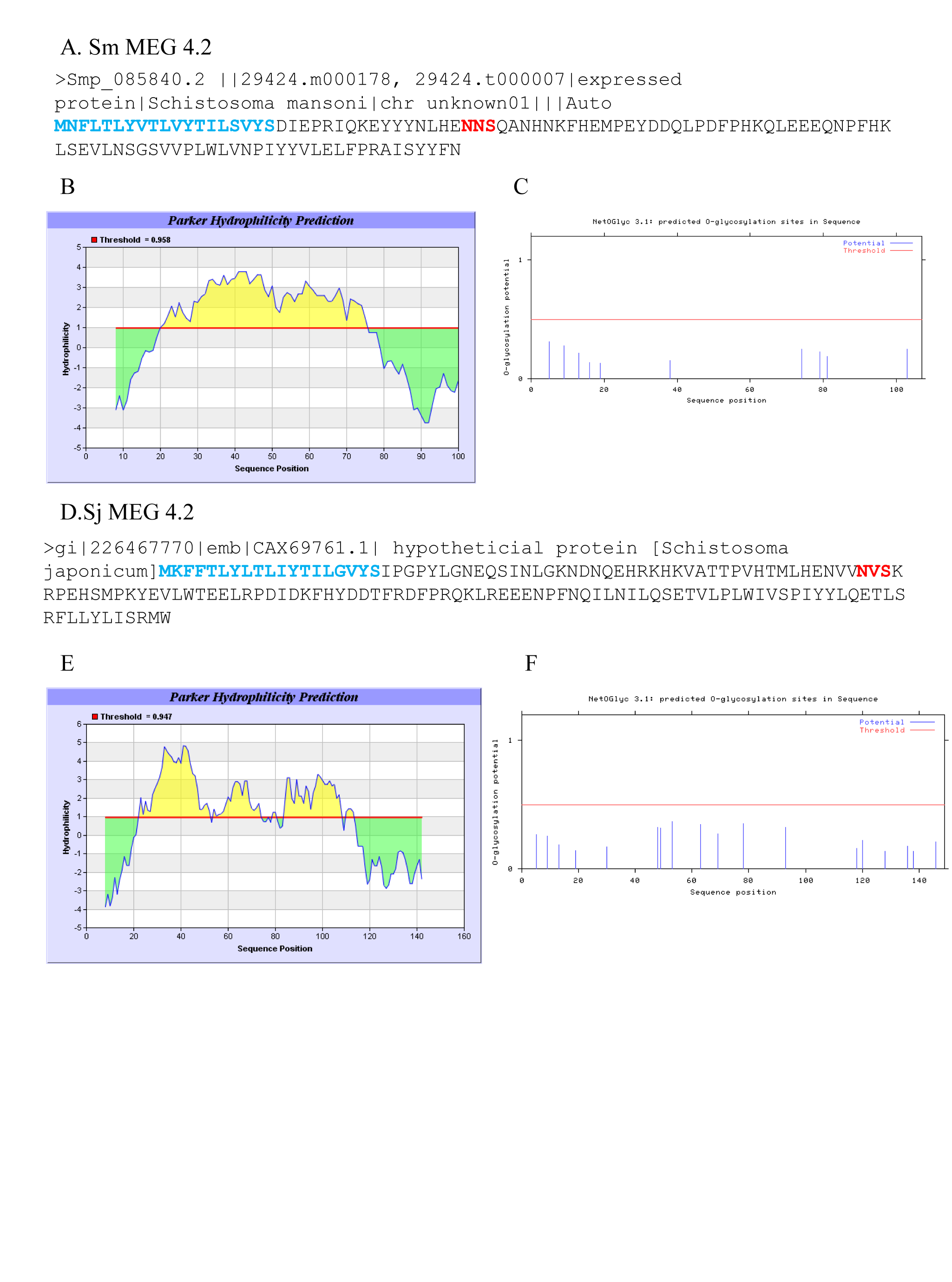

Supplement: Figure S5 — Bioinformatic analysis of MEG-4.2. (A) Amino acid sequence, (B) Parker hydrophilicity plot and (C) O-glycosylation sites predicted by NetOGlyc, of S. mansoni (SmMEG-4.2). (D) Amino acid sequence, (E) Parker hydrophilicity plot and (F) O-glycosylation sites predicted by NetOGlyc, of S. japonicum (SjMEG-4.2). For A and D, the signal peptide is shown in blue and the one N glycosylation site predicted by NetNGlyc is shown in red. (TIF) [file pntd.0002337.s005.tif]

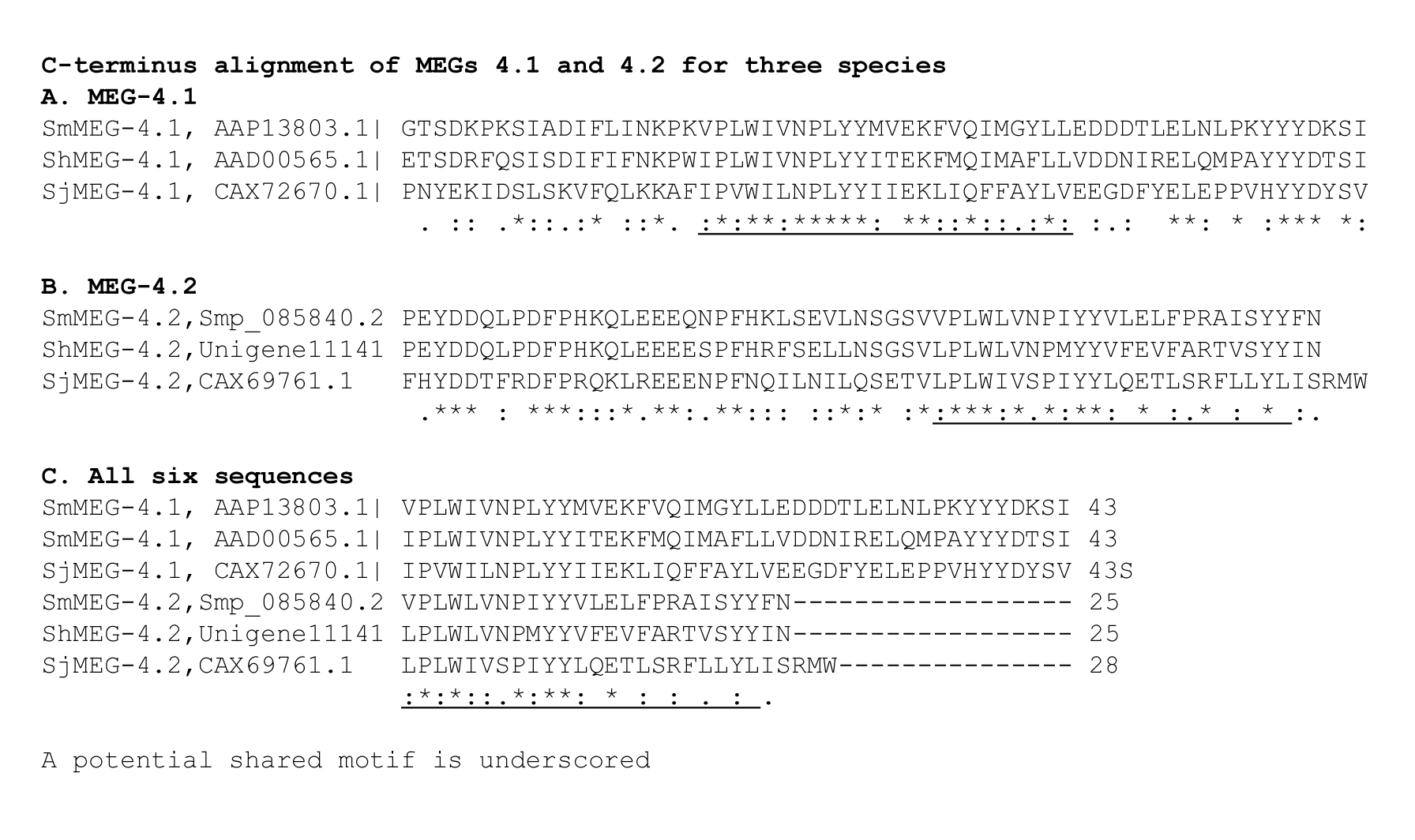

Supplement: Figure S6 — Clustal analysis of the C-terminal region in MEG-4 genes from three species. (A) MEG-4.1, (B) MEG-4.2, (C) The homologous region in all six sequences (underscored in A, B and C). (TIF) [file pntd.0002337.s006.tif]

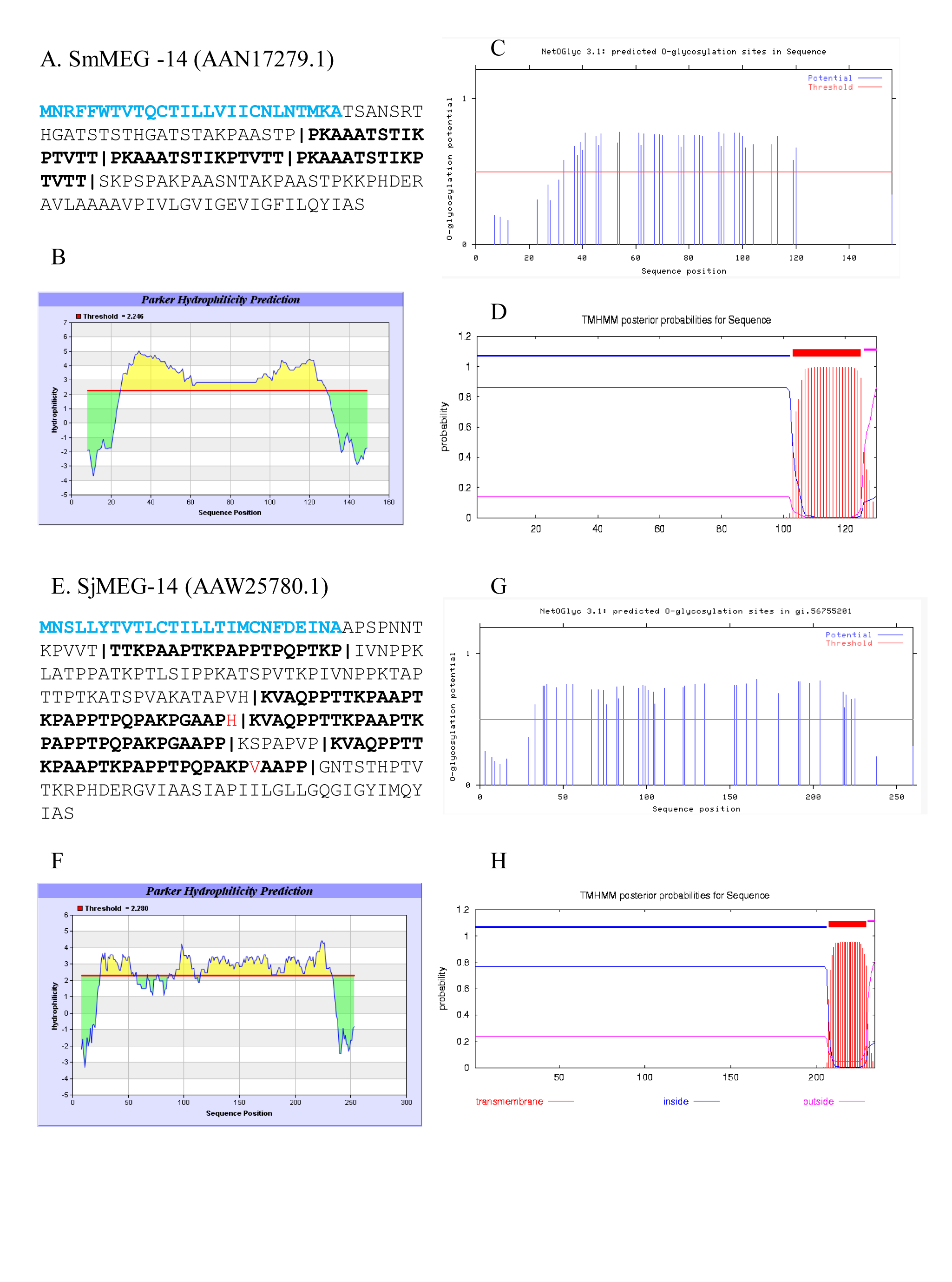

Supplement: Figure S7 — Bioinformatic analysis of MEG-14. (A) Amino acid sequence, (B) Parker hydrophilicity plot, (C) O-glycosylation sites predicted by NetOGlyc and (D) transmembrane region predicted by TMHMM , of S. mansoni (SmMEG-14). (E) Amino acid sequence, (F) Parker hydrophilicity plot, (G) O-glycosylation sites predicted by NetOGlyc and (H) transmembrane region predicted by TMHMM, of S. japonicum (SjMEG-14). For A and E, the signal peptide is shown in blue and repeats are indicated in bold separated by a vertical line. Single amino acid substitutions in the repeats of SjMEG-14 are shown in red. (TIF) [file pntd.0002337.s007.tif]
